# piscesCSM: prediction of anticancer synergistic drug combinations

**DOI:** 10.1186/s13321-024-00859-4

**Published:** 2024-07-19

**Authors:** Raghad AlJarf, Carlos H. M. Rodrigues, Yoochan Myung, Douglas E. V. Pires, David B. Ascher

**Affiliations:** 1 Structural Biology and Bioinformatics, Department of Biochemistry and Pharmacology, University of Melbourne, Melbourne, VIC Australia; 2 Systems and Computational Biology, Bio21 Institute, University of Melbourne, Melbourne, VIC Australia; 3 Computational Biology and Clinical Informatics, Baker Heart and Diabetes Institute, Melbourne, VIC Australia; 4 School of Chemistry and Molecular Biosciences, University of Queensland, Brisbane, QLD Australia; 5 School of Computing and Information Systems, University of Melbourne, Melbourne, VIC Australia

**Keywords:** Drug combination, Machine learning, Graph-based signatures, Synergistic effects, Anticancer drugs

## Abstract

**Abstract:**

While drug combination therapies are of great importance, particularly in cancer treatment, identifying novel synergistic drug combinations has been a challenging venture. Computational methods have emerged in this context as a promising tool for prioritizing drug combinations for further evaluation, though they have presented limited performance, utility, and interpretability. Here, we propose a novel predictive tool, piscesCSM, that leverages graph-based representations to model small molecule chemical structures to accurately predict drug combinations with favourable anticancer synergistic effects against one or multiple cancer cell lines. Leveraging these insights, we developed a general supervised machine learning model to guide the prediction of anticancer synergistic drug combinations in over 30 cell lines. It achieved an area under the receiver operating characteristic curve (AUROC) of up to 0.89 on independent non-redundant blind tests, outperforming state-of-the-art approaches on both large-scale oncology screening data and an independent test set generated by AstraZeneca (with more than a 16% improvement in predictive accuracy). Moreover, by exploring the interpretability of our approach, we found that simple physicochemical properties and graph-based signatures are predictive of chemotherapy synergism. To provide a simple and integrated platform to rapidly screen potential candidate pairs with favourable synergistic anticancer effects, we made piscesCSM freely available online at https://biosig.lab.uq.edu.au/piscescsm/ as a web server and API. We believe that our predictive tool will provide a valuable resource for optimizing and augmenting combinatorial screening libraries to identify effective and safe synergistic anticancer drug combinations.

**Scientific contribution:**

This work proposes piscesCSM, a machine-learning-based framework that relies on well-established graph-based representations of small molecules to identify and provide better predictive accuracy of syngenetic drug combinations. Our model, piscesCSM, shows that combining physiochemical properties with graph-based signatures can outperform current architectures on classification prediction tasks. Furthermore, implementing our tool as a web server offers a user-friendly platform for researchers to screen for potential synergistic drug combinations with favorable anticancer effects against one or multiple cancer cell lines.

**Supplementary Information:**

The online version contains supplementary material available at 10.1186/s13321-024-00859-4.

## Introduction

Cancer, a heterogeneous group of disorders, remains one of the leading causes of death globally, accounting for the deaths of almost 10 million people in 2020 [1]. According to recent data, the number of cancer deaths in the United States will reach 609,820 in 2023, equivalent to about 1670 deaths per day [2]. Consequently, intense research measures are continued to design new effective anticancer treatments.

Therapy resistance and consequent tumour relapse are significant contributors to this disease’s global burden. Cancer drug resistance is a multifactorial problem caused by genetic variability and nongenetic and epigenetic mechanisms, contributing to tumour heterogeneity [3].

While standard monotherapies have made notable advancements in cancer treatment, their effectiveness is greatly restrained by the acquired drug resistance of tumour cells. In light of this challenge, exploring synergistic combinations of FDA-approved cancer drugs has emerged as a promising strategy [4]. Administering combination therapies with a synergistic effect (i.e., when the cumulative therapeutic effect of both drugs exceeds the additive impact of monotherapy) instead of single-drug treatments offers great benefits in overcoming drug resistance, enhancing efficacy, and lowering adverse side effects and toxicity in cancer therapy. Furthermore, the utility of combination therapies extends beyond cancer treatment, being frequently employed to tackle a variety of complex diseases such as, infectious diseases [5], cancer [6, 7] and hypertension [8].

While synergistic drug cocktails generally provide significant treatment benefits, especially in cancer where multiple molecular pathways can be altered, identification of synergistic combinations has progressed slower, with significant scientific, economic, legal, and regulatory barriers [9]. Consequently, there is a pressing need to identify potential synergistic drug combinations for particular cancer types that could enhance synergistic benefits and reduce the adverse effects of anticancer treatments.

The discovery of traditional drug combinations is primarily based on clinical trials and experience [10]. With the expansion of high-throughput screening strategies, researchers can identify synergistic combinations by carrying out in vitro experiments at significant expense. In silico methods, such as machine learning approaches, present the possibility of effectively prioritizing drug combinations for further experimental and clinical validation. By leveraging large datasets and advanced algorithms, machine learning offers a promising approach to discovering novel treatment strategies that can overcome drug resistance and enhance therapeutic efficacy [11].

Several computational approaches have been developed to identify anticancer synergistic drug combinations, using chemical information describing the drugs and molecular details of the cancer cell lines. Both machine learning [12, 13] and deep learning [14–16] algorithms have been developed and trained on up to 60 cancer-specific cell lines to facilitate this process.

Furthermore, advances have been made in disease classification through language model analysis [17], epilepsy seizure recognition [18], and classification of monkeypox skin lesions using convolutional neural networks [19]. Additionally, researchers have harnessed the power of natural language processing to improve disease classification, enabling better diagnosis and treatment [20]. This highlights the broader impact of machine learning in healthcare beyond cancer treatment.

In most cases, a single reference model, the Loewe additivity model, which presumes that drugs act on the same pathway similarly [21], was used as the foundation for drug synergy prediction models developed in the surveyed studies. Nowadays, there is a broad spectrum of well-studied known reference models that are based on distinct chemical and biological assumptions, such as the highest single agent (HSA) [22], Bliss independence [23], zero interaction potency (ZIP) [24], and Loewe additivity [25]**.** Despite this, none of these models is applicable in all cases of drug combinations. This has resulted in model selection becoming a personal choice [3, 21].

While the state-of-the-art approaches mentioned above have shown great promise in predicting synergistic drug combinations, there are some limitations to these methods, such as the need for transcriptomic data of cell lines, including gene expression and copy number, in addition to the requirement of specific pathways or cell lines. In contrast, our approach only requires the chemical structures of both drugs. Another limitation is that most models lack interpretability, which limits their potential for use in clinical settings, an inherent limitation of deep learning techniques that do not readily define and correlate the feature importance of molecular descriptors, such as toxicophores, physicochemical properties, and fingerprints, to drug action in cells.

**Fig. 1:**
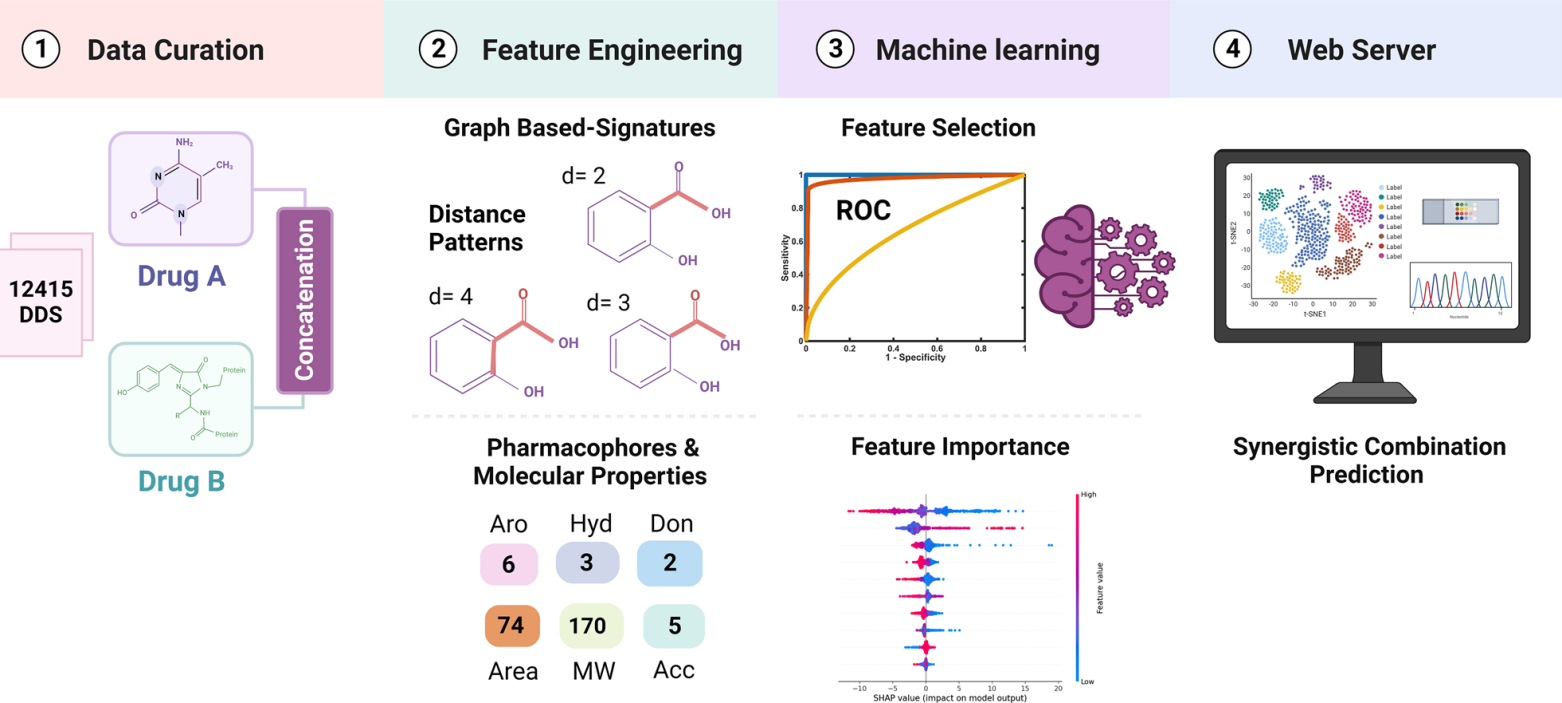
piscesCSM workflow. Our proposed method is divided into four main phases. **1** data curation, the drug-drug synergy (DDS) data was acquired from O'Neil et al. for six different tissue types (39 cancer cell lines); **2** feature engineering, which involved calculating two classes of features: (i) graph-based signatures, that encode small molecules geometry and physicochemical properties, and (ii) general molecular properties and pharmacophores; **3** these were then utilized for training and testing models via supervised learning, with feature selection conducted for model optimization; **4** best-performing models were implemented through an easy-to-use web interface

Prior studies have demonstrated that using the graph-based signature approach efficiently models small molecule properties, ranging from pharmacokinetics and toxicity [26–30] to bioactivity [31–36]. Exploiting this concept, we propose a new machine learning tool, piscesCSM (Fig. 1), which can accurately predict synergistic drug combinations against one or multiple cancer types over different cell lines.

### Problem statement

Cancer remains a leading cause of mortality worldwide, with therapy resistance and tumor relapse posing significant challenges in treatment. Current standard monotherapies have limitations due to acquired drug resistance, highlighting the need for novel anticancer treatment strategies. Machine learning algorithms present a promising avenue for addressing this challenge by providing a more accurate and efficient way of predicting synergistic drug combinations.

### Research gap

While combination therapies are a promising strategy to overcome drug resistance and enhance treatment efficacy, identifying synergistic drug combinations, particularly for specific cancer types, can be challenging. Current methods for predicting synergistic drug combinations may lack accuracy, interpretability, or applicability across different types of cancer.

The primary contributions of this paper are outlined below:

- We proposed piscesCSM, an ML-based model that can accurately predict drug pairs with possible synergistic effects against one or multiple cancer cell lines.
- We utilized the comprehensive O'Neil synergistic drug pairs dataset, ensuring the robustness of our findings across different types of cancer and the model's applicability across various contexts.
- We developed tissue-specific predictive models and demonstrated piscesCSM's performance across different tissue types.
- We explored the interpretability of piscesCSM and demonstrated crucial chemical aspects of drug combinations. This led to improved understanding and trust in the model's predictions.
- We have made piscesCSM freely available as a web server and API for researchers to use and integrate with cheminformatics pipelines to screen potential synergistic drug combinations.

## Materials and methods

### piscesCSM Architecture: modeling synergistic drug combinations

Combination therapies offer significant potential for cancer treatment. We have developed a machine-learning framework for identifying synergistic drug pairs from various combinations. Figure S1 illustrates the overall structure of our proposed model for drug combinations. The architecture of piscesCSM can be summarized as follows:

- **Input data:**
  - Datasets containing drug pairs are loaded and processed to create a comprehensive dataset comprising drug combinations and their corresponding labeling, i.e. antagonistic or synergistic.
- **Feature Engineering Module:**
- **Graph-based signatures**:
  o Computes graph-based signatures capturing geometric and physicochemical properties of each drug individually.
- **Complementary physicochemical properties:**
  o Utilizes RDKit cheminformatics library to compute additional physicochemical properties.
- **Concatenation of features:**
  o Drug combination feature vectors are obtained by combining graph-based signatures with complementary physicochemical properties for each drug pair.
- **Machine Learning Module:**
  - Multiple algorithms for classification:
    o Random Forest
    o Extremely Randomized Trees
    o Gradient Boosting
    o k-Nearest Neighbors
    o Extreme Gradient Boosting
    o Explainable Boosting Machine (EBM)
    o Generalized Additive 2 Model (GA2M)
    o Each algorithm is trained on the concatenated feature vectors to predict the synergy of drug combinations.
  - Hyperparameters optimization:
  - Grid search approach to tune hyperparameters.
  - Assessing performance improvement with stratified cross-validation.
- Greedy Feature Selection Module:
  - Bottom-up greedy feature selection technique:
    o Starts with an empty set of features.
    o Iteratively adds one feature at a time based on performance improvement evaluated using cross-validation.
    o Continues until reaching a predefined number of features or maximum performance.
- Model Evaluation Module:
  - Evaluation metrics include:
    o Accuracy
    o Matthew's Correlation Coefficient (MCC)
    o Precision
    o Area under the ROC curve (AUC)
    o Balanced accuracy
    o Recall
    o F1 Score
  - SHapley Additive exPlanations (SHAP) analysis:
    o Assess feature importance and provide post-hoc justification of model decisions.
- Web Server Development Module:
  - Front end:
    o Developed using Materialize framework for user interface design.
  - Back end:
    o Implemented in Python with the Flask framework to handle requests and responses.
    o Integrating software tools for molecule visualization and format conversion (e.g., Kekule.js, SmilesDrawer, Open Babel, RDKit).
  - Deployment:
    o Hosted on a Linux server running Nginx for accessibility and usability.

Our proposed model architecture incorporates feature engineering, machine learning, feature selection, evaluation, and web server development to predict synergistic drug combinations for cancer treatment. Figure S2 presents a flow chart (pseudocode) encapsulating the key steps of piscesCSM.

### Data curation of anticancer synergistic drug combination

A number of large-scale sets of synergistic drug pairs have been published, two of which have been used in this study. These include O’Neil et al. [37], which contains more than 20,000 pairwise drug synergy scores across 38 approved and experimental drugs. In this way, the performed oncology combination screening covered 83% of the possible two-drug combinations. AstraZeneca [38] have released data from their drug pair experiments, including 11,576 investigations of 910 drug pairs tested on 85 cancer cell lines with molecular-related data. The data mentioned earlier offers the potential to assess computational approaches to predict novel drug combinations.

Here we have trained and validated piscesCSM on an anticancer synergistic drug combination dataset obtained from [37, 38]. Most drug combinations in O’Neil et al.’s data had Loewe additivity values that ranged from − 60 to 60. According to the Loewe additivity model, any synergy score above 0 is considered synergistic. We applied a synergy score of 10 as a threshold to binarize the synergy scores, resulting in a dataset incorporating 12,415 drug-drug combinations (6,300 antagonistic and 6,115 synergistic drug pairs), involving 36 anticancer drugs screened against 31 cell lines originating from 6 different tissue types (See Supplementary Data Set 1 and 2).

For evaluating the generalization and predictive performance of piscesCSM classification models, the datasets were split into non-redundant training (80%) and blind test (20%) sets. The drug similarity of each pair of drugs was determined by clustering the drug pairs based on Morgan/Circular fingerprints with the Tanimoto coefficient (at a 0.6 similarity level) using the Butina algorithm applied via the RDKit library [39]. This was done to ensure that similar drug pairs were present in the training or testing sets. All datasets employed in the current study are available at https://biosig.lab.uq.edu.au/piscescsm/data.

### Feature engineering

We adopted our well-established graph-based signatures approach to model chemical entities by describing their geometry and physicochemical properties. Our method proposes an intuitive graph representation of a compound that can be obtained by representing atoms as nodes (labelled based on their pharmacophoric properties) and their covalent bonds as edges. By altering a distance cut-off, cumulative distributions of distances are generated, forming a concise and efficient representation of the chemical entities. This information is then employed to train and test predictive models applying supervised learning. We have previously introduced the concept of graph-based signatures to describe protein structure geometry and the molecular interactions with their binding partners as graphs [40–54]. These were successfully employed and adapted to train and test various machine learning models, including the prediction and optimization of pharmacokinetics and toxicity properties [26, 27, 30], in addition to the identification of bioactive compounds with anticancer properties [31].

Here, we adapted this concept to model drug combinations. We calculated these signatures for each drug individually; in this way, each drug was represented by a vector of 264 components, and then the features of each drug combination were concatenated into a vector of 526 input features.

Complementary physicochemical properties were also calculated using the RDKit cheminformatics library [39]. A list of the features explored in our study, as well as the characteristics and composition of the dataset used, are detailed in Tables S1 and S2, respectively.

### Machine learning approaches and model evaluation

We trained and evaluated several learning algorithms to obtain classification models for predicting synergistic drug combinations. These included Random Forest, Extremely Randomized Trees, Gradient Boosting, *k*-Nearest Neighbors, and Extreme Gradient Boosting, using the implementation available on the Scikit-learn library [55]. Furthermore, using the open-source Python module InterpretML [56], a glass-box model known as Explainable Boosting Machine (EBM), an inherently interpretable strategy, a class of Generalized Additive 2 Model (GA2M), was evaluated. In interpretable machine learning models, the goal is to provide reasoning behind prediction in which biological insight can be gained and help identify highly predictive variables (features), biases, and errors.

The hyperparameters employed to train the piscesCSM model, along with the model's predictive performance both before and after hyperparameter optimization, are presented in Tables S3 and S4, respectively. A grid search technique available via the Scikit-Learn library [55] was adopted for Hyperparameters optimization; a notable performance improvement was observed. The hyperparameters were tuned using stratified fivefold cross-validations.

In addition to hyperparameters tuning, a bottom-up greedy feature selection procedure [57] was utilized to reduce the redundancy, noise, and model complexity. In this approach, the feature set begins without any features and is built up one by one through iteration. This method uses a tenfold cross-validation procedure on a machine learning algorithm to evaluate all features (besides those already selected) to include one in the feature set. Each feature is assessed based on Matthew's correlation coefficient in the classification task. The best-performing feature is then incorporated with the current set at this point. Finally, Matthew's correlation coefficient was also used to determine the models with the best performance based on greedy feature selection. Notably, Matthew's correlation coefficient was favored as it enables choosing models that would be resilient to class imbalances.

After greedy feature selection, the Extremely Randomized Trees presented the best predictive performance on fivefold cross-validation. Predictive performance was evaluated using accuracy, Matthew’s Correlation Coefficient (MCC), precision, the Area under the ROC curve (AUC), balanced accuracy, F1 score and recall. The summary plot method of SHapley Additive exPlanations (SHAP) [58] was utilized to evaluate the final models’ features’ importance and provide a post-hoc justification of our models’ decision.

### Web server development

The web server front end was developed via Materialize framework version 1.0.0. The back end was built in Python 2.7 using the Flask framework (version 0.12.3) and the Scikit-Learn (0.20.3) library [55]. It is hosted on a Linux server running Nginx. The piscesCSM web server integrates many software tools with permissible licenses. The Kekule.js editor [59] is used for drawing molecules and SMILES strings. While molecule depictions can be visualized using SmilesDrawer (version 1.0.10) [60]. The molecular format conversion process uses Open Babel (version 2.4.1) [61] and RDKit cheminformatics library (2017.09.03) [39]. In addition, our developed tool pkCSM [26] is employed to calculate the input molecules' pharmacokinetic properties (users' molecules of interest).

## Results

### Exploring the embedding space of drug synergism for different tissue types

**Fig. 2:**
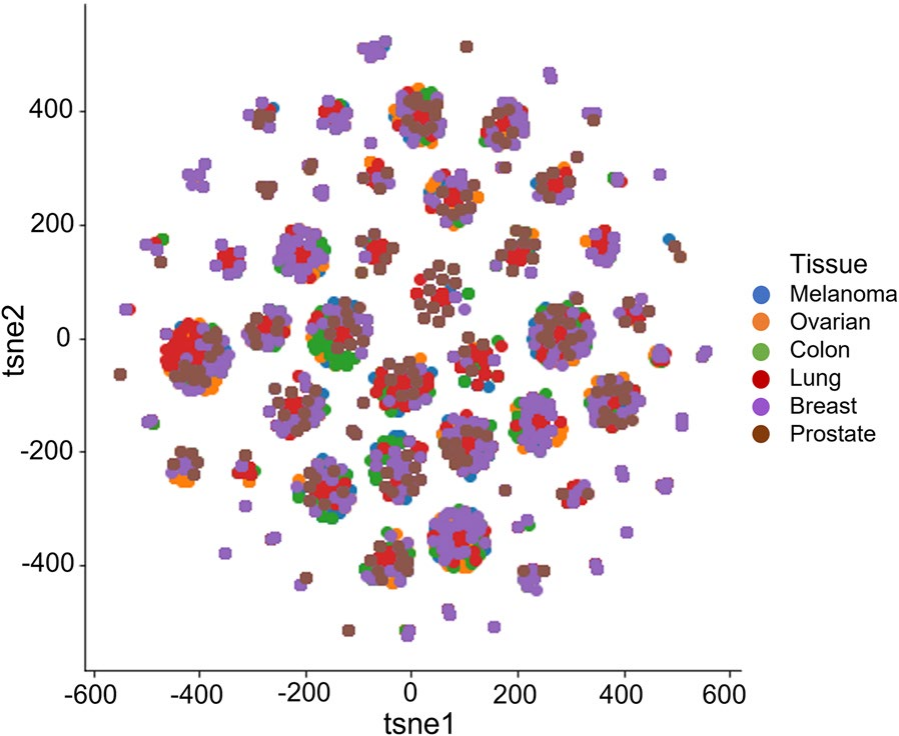
Visualization of distinct tissue types using t-SNE analysis to investigate the embedding space. High-dimensional vector representations are mapped into 2-D space with two t-SNE elements. Different colors denote various tissues

Across our dataset, we curated screening information from six different tissues, including colon, breast, melanoma, ovarian, prostate, and lung (in total 12,415 combination pairs). This raised the question of whether differences in synergistic behavior might vary between tissue types. We, therefore, explored how different tissues clustered based on shared molecular features between combinations. To reflect the relationships among various tissues of origin, we, therefore, conducted a t-Distributed Stochastic Neighbor Embedding (t-SNE) analysis to visualize tissues' high-dimensional representation embedding vectors in a 2D space (Fig. 2). This revealed that most tissue types were clustered together in the 2-D space. This supported the idea for a general analysis and predictive model, with the larger data size providing increased statistical power.

Interestingly, some cell lines originating from the breast tissue were isolated and tended to form isolated clusters, indicating that they may have unique molecular characteristics. This is consistent with earlier work [16], which reported that two breast cancer cell lines are outliers when analyzing drug combination screens. This requires further investigation but has potentially important ramifications, both clinically and within research.

### Exploring properties of synergistic anticancer drug combinations

Using a large-scale oncology screen dataset incorporating the synergy of anticancer compounds for 12,415 drug combinations, we conducted a two-sample Kolmogorov–Smirnov to explore which molecular features correlate with a synergistic anticancer effect. We observed that synergistic combinations tended to involve molecules with more rings, a higher number of rotatable bonds, a slightly greater Logp, and larger Kppa2 values (which is used to estimate the inter-rater reliability of the compounds). Interestingly, drug combination pairs also had a higher frequency of methoxy groups, consistent with previous observations that showed drug combinations containing methoxy groups exhibited synergistic antitumor activity in vitro [62]. Antagonistic drug pairs, in contrast, tended to have a higher frequency of piramide**.** Figure S3 illustrates the leading discriminative features of the synergistic drug pairs compared to antagonistic combinations.

### Predicting anticancer synergistic drug combinations

Combinatorial therapy is a favourable strategy to alleviate drug resistance compared to anticancer monotherapy; therefore, we collected an extensive screening oncology dataset of 12,415 unique drug pairs with experimentally described synergistic effects against multiple cancer types. The acquired data was divided into non-redundant training (80%) and blind test (20%) sets.

Then, we trained classification (5040 antagonistic pairs /4893 synergistic pairs) models using different supervised machine learning algorithms that leveraged graph-based signatures and general physicochemical properties to accurately predict favourable synergistic combinations across multiple cancer lines.

Under stratified fivefold cross-validation, our best-performing extremely randomized trees obtained an overall balanced accuracy of 0.82, AUC of 0.89, MCC of 0.61, a precision of 0.82, F1 score of 0.81 and recall of 0.82 (Figure S4 and Table 1). This was consistent with performance on tenfold and 20-fold cross-validation (Table S5). When we evaluated the predictive performance of our model against a blind test set, it achieved comparable performance (0.81, 0.87, 0.59, 0.82, 0.81 and 0.81 for balanced accuracy, AUC, MCC, precision, F1 score and recall, respectively). This provided confidence that our proposed method generalizes well and can be employed to predict novel synergistic combinations against multiple cancer cell lines. Figure S5 visually presents the confusion matrices, depicting the counts of correctly and falsely predicted samples by piscesCSM, evaluating its classification performance on both cross-validation and blind test sets.

**Table 1:** Comparative performance of piscesCSM and other competitive methods

Method	Five fold Cross-validation					
	ROC AUC	MCC	Balanced Accuracy	Precision	Recall	F1 score
piecesCSM	0.89	0.61	0.82	0.82	0.81	0.81
DeepDDS-GAT [63]	0.90	0.60	0.82	0.82	0.80	0.81
DeepSynergy [15]	0.88	0.57	0.80	0.81	0.75	0.78

The performance of piscesCSM was evaluated and compared with alternative approaches using the dataset developed by O'Neil et al*.* [37], which has been used in many previous approaches, including DeepSynergy [15] and DeepDDS [63] (Table 1). piscesCSM obtained higher recall than all other approaches and outperformed DeepSynergy across all performance measures. Compared to DeepDDS-GAT, piscesCSM obtained stronger results across MCC and recall without significant deterioration of balanced accuracy and precision, while DeepDDS-GAT achieved higher AUC. In addition, when comparing our model performance with the alternative methods on the blind test set, piscesCSM outperformed both approaches, as shown in Table S6**.**

### Exploring piscesCSM tissue-specific predictive performance

Since cancer is more than a single disease and drug-combination treatment has tissue-specific responses, we, therefore, used the graph-based signatures approach to predict synergistic anticancer effects across six distinct tissue types: colon, breast, melanoma, ovarian, prostate, and lung. Please refer to Table S7 for the detailed breakdown of training and testing samples corresponding to each tissue type.

**Fig. 3:**
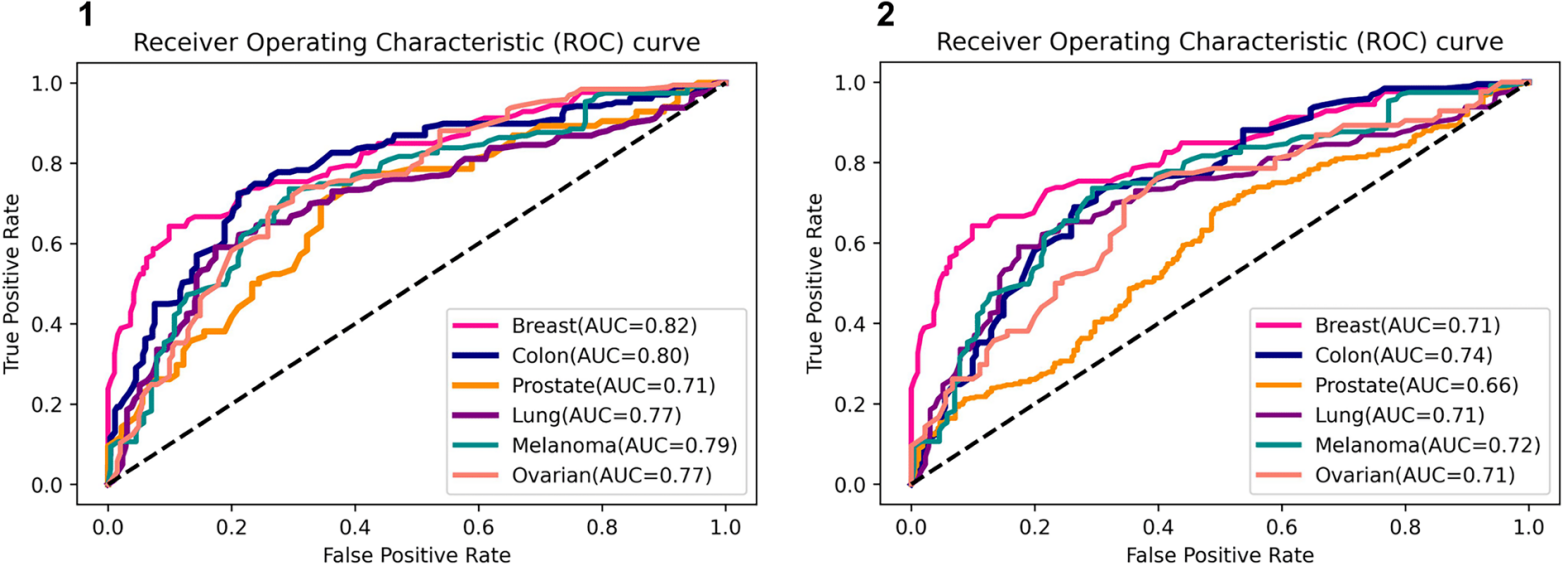
piscesCSM tissue-specific performance depicted as ROC curves. Our predictors accurately classified synergistic drug combinations. **1** Predictive performance of piscesCSM tissue-specific models on cross-validation. **2** Tissue-specific comparative performance on blind test sets

We trained and developed six tissue-specific classification models using supervised learning (categorical outcomes were present in all data sets: synergistic vs antagonistic). The final models obtained AUCs of up to 0.82, and an F1 score of up to 0.80, with MCC and balanced accuracy of up to 0.58 and 0.80, respectively, under tenfold cross-validation; overall, the predictive performance did not differ considerably across distinctive tissues, except for the prostate (Fig. 3-1). Prostate tissue had the lowest performance among all tissues studied. The limited predictive performance could be primarily due to the small number of training samples.

Our tissue-specific models achieved comparable performance across the non-redundant blind test sets, achieving AUCs, MCC, F1 scores and accuracy of up to 0.74(Fig. 3-2), 0.48,0.73 and 0.71, respectively, providing confidence in the generalizability of our approach in all tissue types. The ROC curves for the six tissue-specific predictive models are illustrated in Fig. 3, demonstrating their performance on cross-validation and blind test sets. Additionally, Figures S6 and S7 depict the confusion matrices for these models, showcasing their performance evaluation on cross-validation and blind test sets.

### Performance analysis and comparison on low-redundancy settings

We further evaluated our model's performance under low-redundancy settings by employing three different leave-one-group-out cross-validations schemes, in addition to comparing its performance with the state-of-the-art methods DeepDDS [63] and DeepSynergy [15] (Table 2).

**Table 2:** Comparative performance of piscesCSM on low-redundancy settings, including leave-one-drug-combination-out, leave-one-drug-out and leave-one-tissue-out cross-validations

Method	Leave-drug combination-out				Leave-drug-out				Leave-tissue-out			
	ROC AUC	Balanced Accuracy	MCC	F1 score	ROC AUC	Balanced Accuracy	MCC	F1 score	ROC AUC	Balanced Accuracy	MCC	F1 score
piscesCSM	0.89	0.81	0.63	0.81	0.79	0.79	0.56	0.79	0.88	0.81	0.60	0.81
DeepDDS-GAT [63]	0.90	0.81	0.62	0.81	0.73	0.66	0.48	0.66	0.83	0.74	0.56	0.74
Deep Synergy [15]	0.83	0.77	0.57	0.77	0.71	0.61	0.45	0.61	0.80	0.71	0.52	0.71

The first scheme was leave-one-drug-combination-out, where each drug combination was iteratively used as a test set. piscesCSM performed as well as or better than all alternative approaches (p-value: < 0.05), achieving an AUC of 0.90 and balanced accuracy of 0.81.

A leave-one-drug-out evaluation was also conducted to assess the model’s ability to generalize for unseen drugs, also significantly outperforming alternative methods (p-value: < 0.05), achieving up to 0.18 higher balanced accuracy. A leave-one-tissue-out cross-validation strategy was also adopted by using individual tissues iteratively as test sets. No significant performance deterioration was observed for piscesCSM, which consistently outperformed other methods (Table 2).

**Fig. 4:**
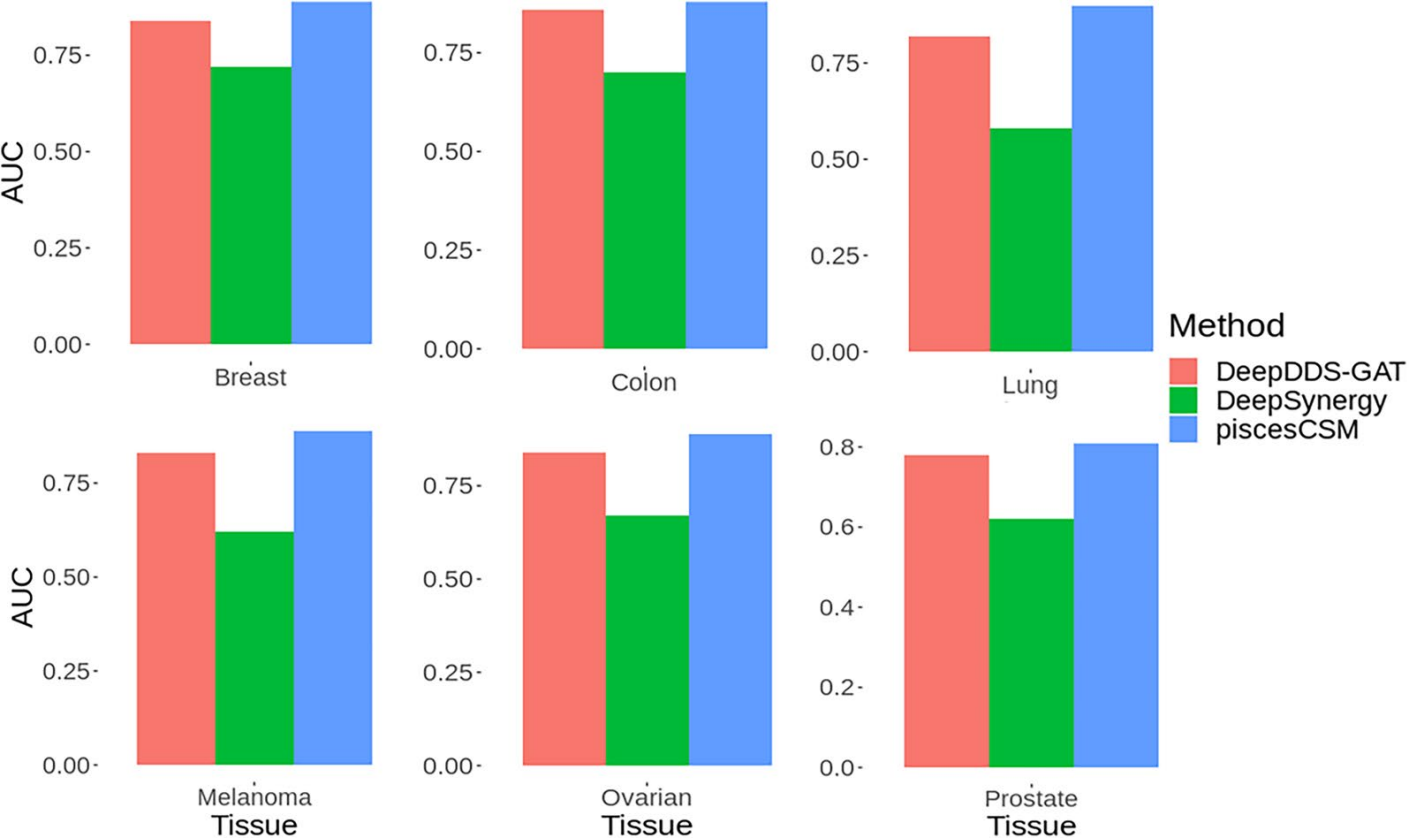
Performance comparison of piscesCSM, DeepDDS-GAT and DeepSynergy on leave-one-tissue-out cross-validation experiments using AUC as the evaluation metric on six distinct tissue types: breast, colon, lung, melanoma, ovarian and prostate

Further, Fig. 4 depicts the ROC AUC values of our model, DeepDDS-GAT, and DeepSynergy on six tissue types: breast, colon, lung, melanoma, ovarian and prostate. It is noted that piscesCSM outperformed other competitive approaches on leave-one tissue-out cross-validation (using a Wilcoxon signed rank-sum test, p-value: < 0.05) with ROC AUC values of 0.89, 0.88, 0.90, 0.89, 0.89 and 0.81, respectively.

### Evaluation using the AstraZeneca independent data

To further evaluate the generalizability of our approach, we utilized an independent test set initially published by AstraZeneca [38]. The data incorporates 668 distinctive drug pair–cell line combinations, including 57 drug pairs (see Supplementary Data Set 3) and 24 cell lines (Table S5). Interestingly, when we explored the chemical diversity of drug pairs between and within our training and the AstraZeneca independent blind test sets using Tanimoto similarity, we found that these datasets had Tanimoto similarity indices of 0.117 and 0.154, respectively, implying a high level of chemical diversity in the applied dataset (Figure S8). piscesCSM correctly identified 429 of the drug combination pairs, followed by DeepsDDS-GAT [63], correctly predicted 406, compared to only 317 by the state-of-the-art approach DeepSynergy [15] (p-value: < 0.05) (Table 3 and Figure S9). Figure S10 illustrates the confusion matrices for the three methods, providing a detailed breakdown of the correctly and falsely predicted samples.

**Table 3:** Performance comparison of piscesCSM with DeepSynergy and DeepDDS-GAT on the AstraZeneca independent blind test

Method	ROC AUC	MCC	Balanced accuracy	Precision	Recall	F1 score
piscesCSM	0.66	0.47	0.64	0.82	0.63	0.71
DeepDDS-GAT [63]	0.65	0.42	0.59	0.78	0.63	0.70
DeepSynergy [15]	0.56	0.37	0.53	0.76	0.40	0.51

### Understanding chemotherapeutic synergism through interpreting feature importance in piscesCSM model

Interpreting a prediction model’s output correctly is essential, as it provides a better understanding of the process being modelled as well as how a model could be refined, consequently supporting clinical decision-making. Therefore, to interpret the decisions behind piscesCSM tissue-specific predictions, better understand the predictive models and hopefully shed light onto what makes an effective synergistic drug combination against different cancer tissue types. We explored the interpretability of our piscesCSM tissue-specific models in two different scenarios at a global interpretability level and a post hoc prediction level.

To begin with, a highly interpretable glass box model- the Explainable Boosting Machine (EBM)- [56] was employed to understand overall feature importance and provide a global explanation (what the final models have learnt broadly) of the features utilised by the tissue-specific models. The ROC curves of the best EBM tissue-specific models are illustrated in Figures S11 and S12. By calculating the average absolute contribution of features in predicting training data for each tissue-specific classifier, the overall importance ranking (global explanation) was determined.

Figures S13-S15 show the global explanations of the tissue-specific EBM models. The global interpretability analysis showed that the most important variables for breast and colon-specific models were distance patterns that involve pairs of hydrophobic and acceptor atoms within four bonds (i.e., Hydrophobe: Hydrophbe-4.00_drug_B, and Acceptors: Hydrophbe-4.00_drug_B). In comparison, the most important variables for Melanoma, prostate and Lung-specific models included general molecular descriptors, such as MOE-like descriptors of molecular surface area (such as PEOE_VSA_1, PEOE_VSA9_drugA, and SMR_VSA5). Similarly, topological descriptors, incorporating Chi1n and Chi0n, were the first two most predictive variables in the Ovarian-specific model.

We have further investigated the features' interpretability of the top most predictive variables in the tissue-specific models as a part of the global explanation analysis. The plots of the features' interpretability for the tissue_ specific models are depicted in Figures S16–S18.

Interpretability plots can be interpreted as two-dimensional risk profiles, where the horizontal axis is the actual value of each feature, and the vertical axis represents the risk score (upper graphs in Figures S16-S18). The values distribution of the feature is also reported in the bottom graphs in Figures S16-S18. An increase in a feature risk score above zero indicates that the feature contributes to the classification in the positive direction (synergistic). In contrast, a feature risk score below zero suggests a contribution in the negative direction (antagonistic). For example, the plot of interpretability for the most important variable in the colon-specific model, which depicts distance patterns incorporating hydrophobic atoms pairs within four bonds in drug B (Figure S16), shows this feature as having values higher than 2.5, denoting a synergistic combination. In contrast, actual values between 0 and 2.3 contribute to predicting the antagonistic combination (effect).

Likewise, the interpretability plot (Figure S17) for the molecular surface descriptor for drug B (SMR_VSA5), the most important variable in the lung-specific model, demonstrates that actual values between 13 and 21 contribute to the classification of anticancer synergistic combination. Conversely, values between 0 and 12 contribute to the prediction of an antagonistic combination.

Furthermore, a post-hoc analysis was conducted employing the Shapley Additive exPlanations (SHAP) [58] method to understand individual feature contributions to the model outcomes.

SHAP feature importance values were calculated for each tissue-specific predictive model (Figures S19–S24). The values calculated by the SHAP plot indicate the distribution of the impact of respective features on the model’s result. Generally, the top features on each plot contribute more to the model prediction than those at the bottom.

Noticeably, for most models, the strongest contributing features for predicting synergistic anticancer effects were the general physicochemical properties of the compounds, including the number of Heavy atoms and descriptors of molecular surface area (PEOE_VSA and SlogP_VSA), as well as the topological descriptors of the compounds (e.g., Chi4v). In addition, the graph-based signature representations of the molecules were demonstrated to play a vital decision role, particularly highlighting the presence of aromatic groups, such as the number of pyridine rings, in line with a previous study that demonstrated compounds incorporating pyridine-derivatives exhibited synergistic antitumor effects in vitro [64]—furthermore, distance-based patterns involving donor atoms (e.g., Donor: Hydrophobe-2.00_drug_B). Interestingly, the colon-specific model differentiated from the other models, incorporating fragment-matching descriptors such as fr_ester, fr_aniline, and fr_piperzine.

### piscesCSM web server

To help guide researchers to screen for novel anticancer synergistic combinations more efficiently, we have implemented piscesCSM through an easy-to-use web server and API and made it freely available at https://biosig.lab.uq.edu.au/piscescsm/. To predict synergistic anticancer drug combinations, users can submit their molecules of interest to the server either as a single smile string or as a batch file by submitting molecules as SMILES strings. Additionally, users can calculate the pharmacokinetic properties of their molecules of interest by employing the pkCSM tool [26] (Figure S25).

## Discussion

In this study, we introduced piscesCSM, a machine-learning-based method that combines graph-based signatures and physicochemical properties to provide better predictive accuracy and interpretability for predicting synergistic drug combinations.

Our study demonstrates the prospect of machine learning to transform cancer treatment strategies. Our proposed model, piscesCSM, leverages large-scale datasets of synergistic drug combinations to predict such combinations accurately and reliably across multiple cancer types and cell lines. This can potentially guide the development of more effective and personalized cancer therapies.

Furthermore, we have developed a user-friendly web server to facilitate easy access to our predictive model. Thereby enabling researchers and healthcare professionals to screen for potential synergistic drug combinations efficiently, accelerating the translation of computational findings into clinical practice.

## Limitations

Despite our study’s promising results, some limitations should be acknowledged. Firstly, our predictive model relies on a limited dataset of anticancer drug combinations, which may not encompass the full spectrum of potential interactions or account for all relevant factors influencing drug synergy. Incorporating additional datasets and refining our model with real-world clinical data can enhance its predictive performance and generalizability.

Furthermore, factors such as data availability, the heterogeneity of cancer types, and variability in patient responses to treatment may limit the applicability of our model. Fostering interdisciplinary collaborative efforts and ongoing refinement of our model through user feedback is essential to addressing these limitations and optimizing cancer therapy.

## Conclusion

Computational approaches have been developed and employed over the years to assist prediction and prioritization of possible synergistic drug combinations, though presented limited performance and interpretability. Here we proposed a novel approach to predict synergistic drug combinations against one or multiple cancer types over different cell lines, piscesCSM, leveraging the concept of graph-based signatures. We demonstrated our model not only outperformed alternative approaches on multiple independent blind test sets but presented consistent performance, even on low-redundancy settings. This provides confidence in the model’s generalization capabilities for novel drug combinations, drugs, and tissues.

In contrast with alternative black-box approaches, we have assessed the rationale behind model predictions, interpreting feature importance. This showed that simple physicochemical properties (mostly surface area) and graph-based signatures could accurately predict chemotherapy synergism.

As larger publicly available synergy datasets become available, piscesCSM could be further enhanced and used in other fields where drug combinations play a vital role, including antifungal [65], antiviral [66], and multidrug synergy prediction [67]. We leveraged graph-based signatures for modelling small molecule physicochemistry of an extensive screening oncology dataset data set of drug pairs with experimentally described synergistic effects and illustrated their efficacy. We anticipate piscesCSM will be an invaluable in silico tool for identifying potential synergistic drug combinations and guiding in vitro and in vivo rational experimental validation of future combination therapies.

In terms of future work, several potential avenues could help shape therapeutic strategies and predict the most effective drug combinations. One of these avenues involves integrating and leveraging diverse omics data types, such as genetics, gene expression, proteins, and metabolites. Analyzing these data types can provide a comprehensive understanding of cancer biology and drug response mechanisms, as well as how drugs interact at the molecular level, helping identify the best drug combinations.

Artificial intelligence, particularly deep learning algorithms, is another critical factor in predicting drug combinations. These advanced algorithms can identify complex patterns in data, which is ideal for capturing the nonlinear relationships within our bodies. By leveraging these algorithms, more accurate predictions about drug interactions and the identification of novel synergistic drug pairs can be achieved.

Moreover, precision medicine approaches tailored to individual patient profiles are promising for optimizing treatment outcomes and minimizing adverse effects, ultimately leading to a new era in oncology treatment.

### Supplementary Information

Additional file 1.

Additional file 2.

Additional file 3.

Additional file 4.

## Data Availability

All models developed and code are available at https://biosig.lab.uq.edu.au/piscescsm/ and https://bitbucket.org/ascherslab/piscescsm_standalone/src/master/, and all data used in this study are available in Supplementary Information and https://biosig.lab.uq.edu.au/piscescsm/data
