# Supplementary material for: piscesCSM: prediction of anticancer synergistic drug combinations
Source: J Cheminform. 2024 Jul 19;16:81. doi: 10.1186/s13321-024-00859-4 (PMC11264925; doi:10.1186/s13321-024-00859-4)
Supplement: Supplementary file 1 — Additional file 1. [file 13321_2024_859_MOESM1_ESM.docx]

**Supplementary material**

**piscesCSM: prediction of anticancer synergistic drug combinations**

Raghad AlJarf^1,2,3^, Carlos H. M. Rodrigues^1,2,3,4^, Yoochan Myung^1,2,3,4^, Douglas E. V. Pires^1,2,3,4,5^, David B. Ascher^1,2,3,4^

1 Structural Biology and Bioinformatics, Department of Biochemistry and Pharmacology, University of Melbourne,Melbourne, Victoria, Australia

2 Systems and Computational Biology, Bio21 Institute, University of Melbourne,Melbourne, Victoria, Australia

3 Computational Biology and Clinical Informatics, Baker Heart and Diabetes Institute, Melbourne, Victoria, Australia

4 School of Chemistry and Molecular Biosciences, University of Queensland, Brisbane, Queensland, Australia

5 School of Computing and Information Systems, University of Melbourne, Melbourne, Victoria, Australia

**Figures**

**
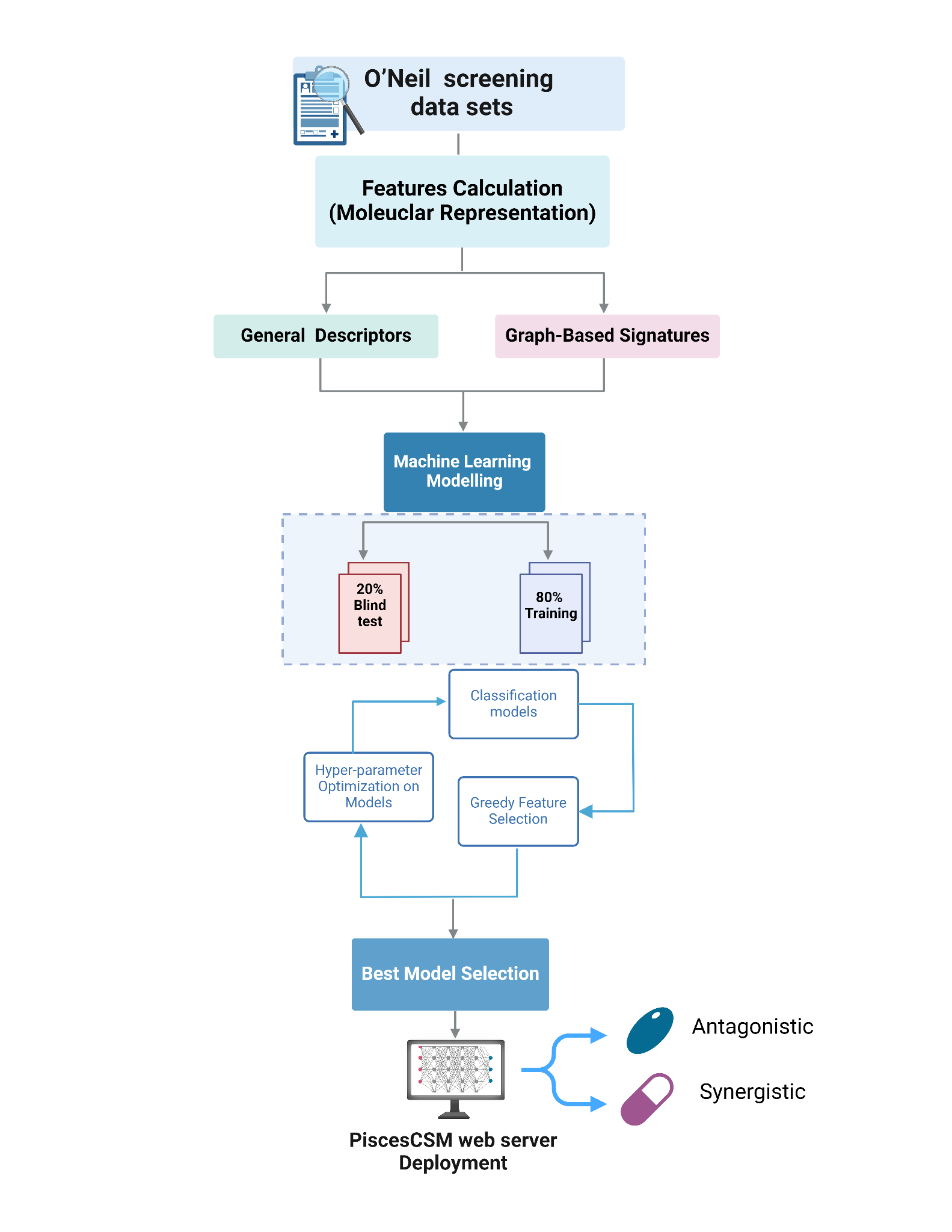
**

**Figure S1**.The proposed model's general architecture is presented as a flow chart, outlining the main methodological steps followed by piscesCSM.


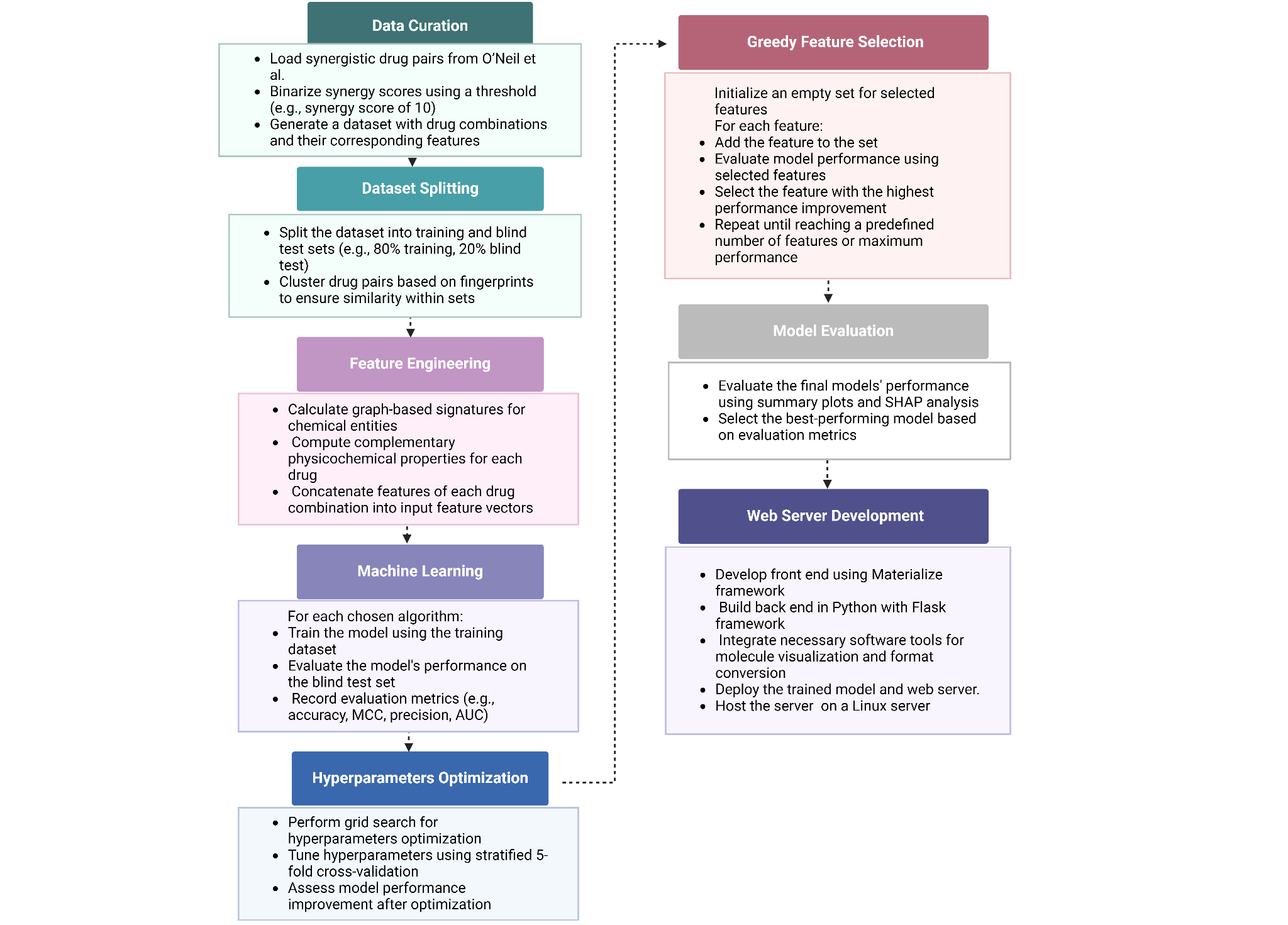


**Figure S2**. A flowchart showing the main steps of the proposed model-piscesCSM.


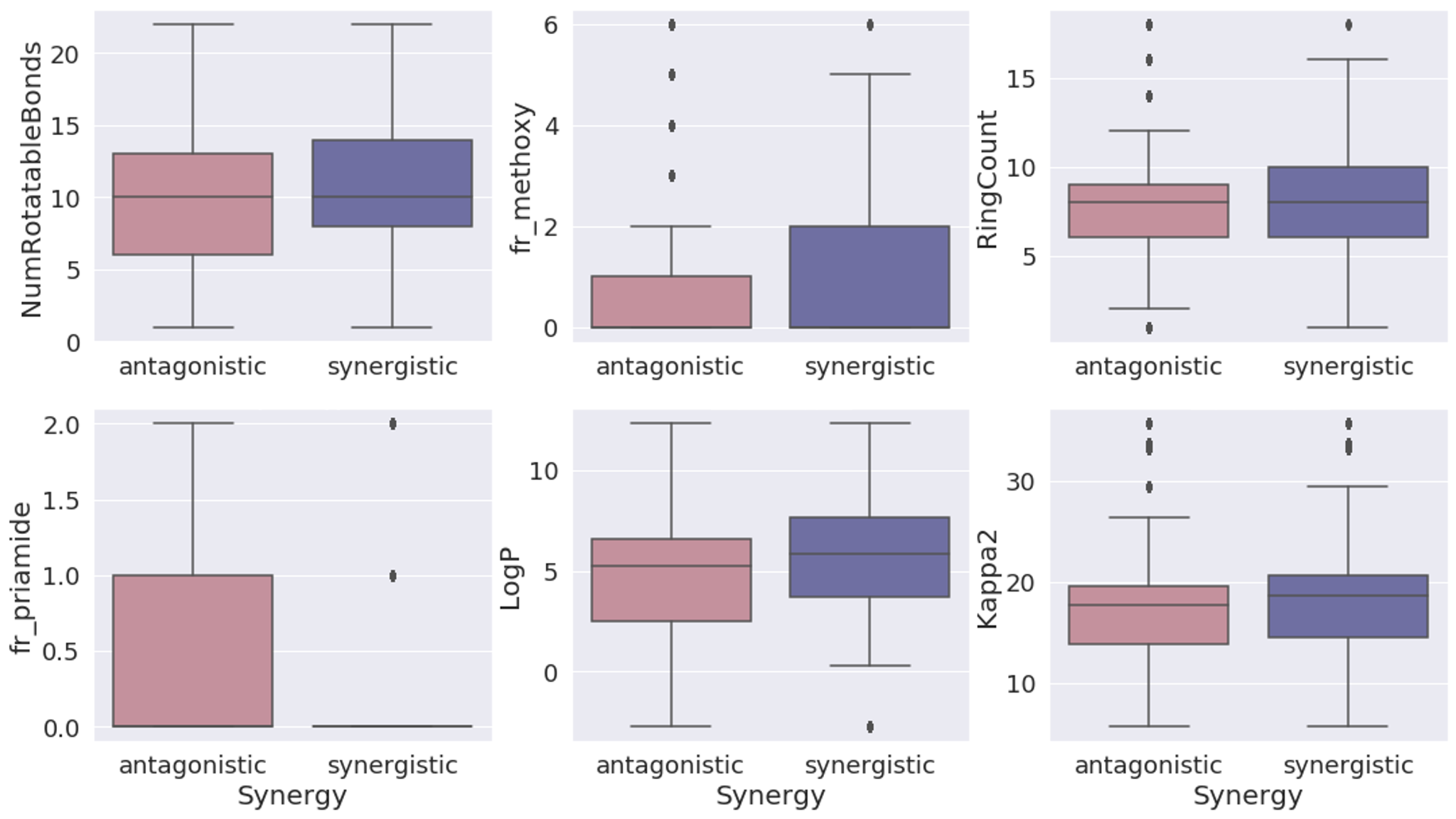


**Figure S3**. Top discriminative features of synergistic drug pairs compared to antagonistic combinations.


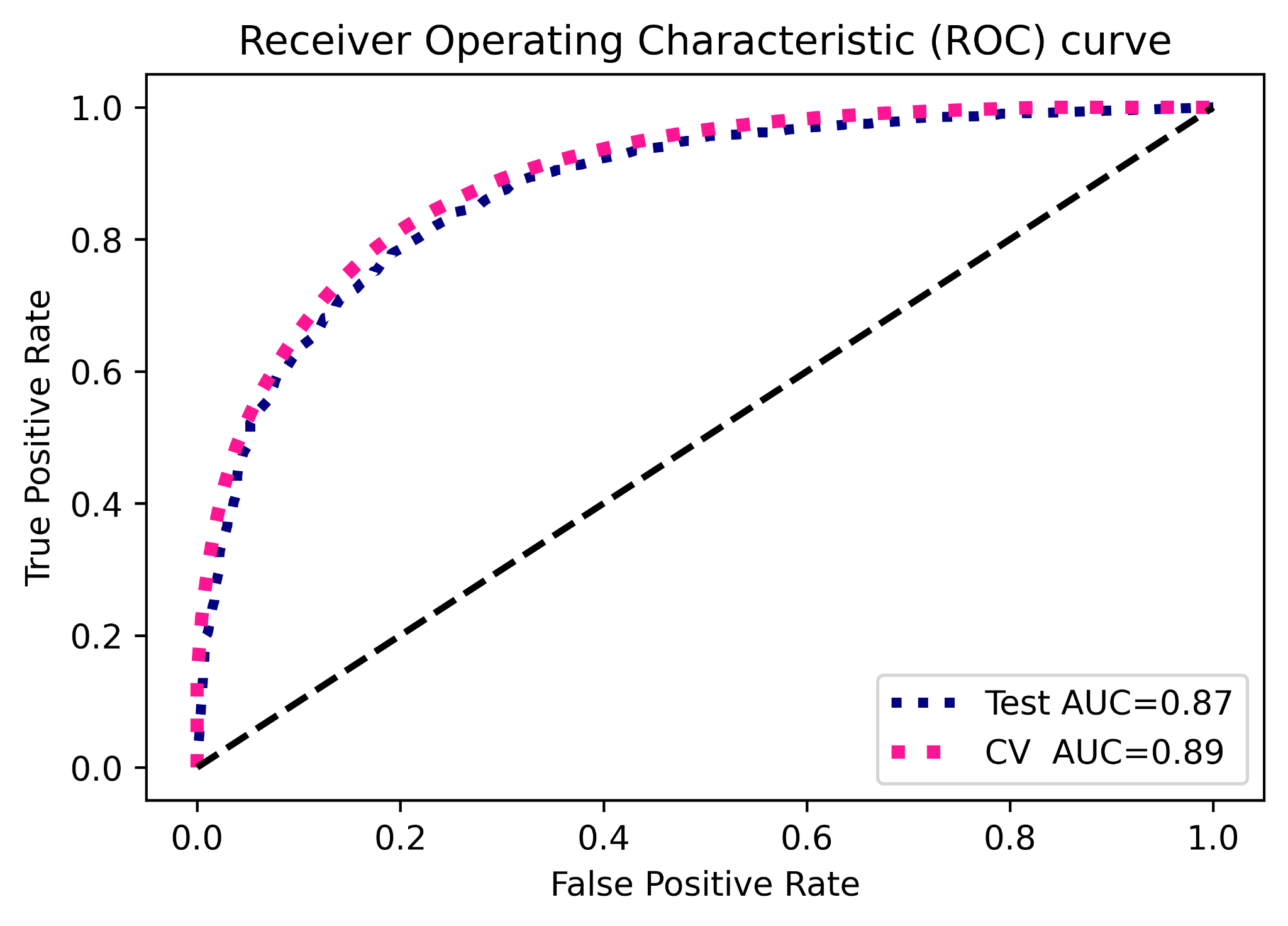


**Figure S4.** Performance of piscesCSM classification model. Our predictor accurately identified synergistic drug pairs with AUC > 0.86 on cross-validation and blind tests.

**
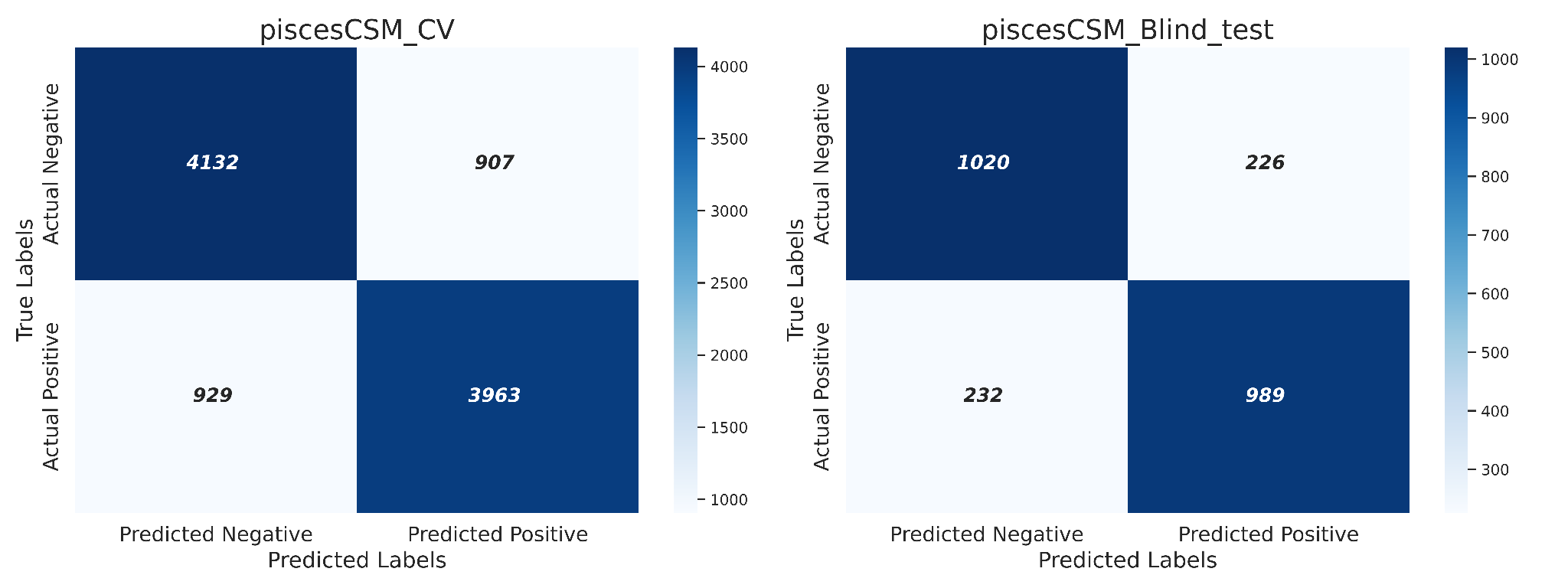
**

**FigureS5.** The confusion matrices for piscesCSM on both the 5-fold cross-validation (left) and blind test sets (right).

**
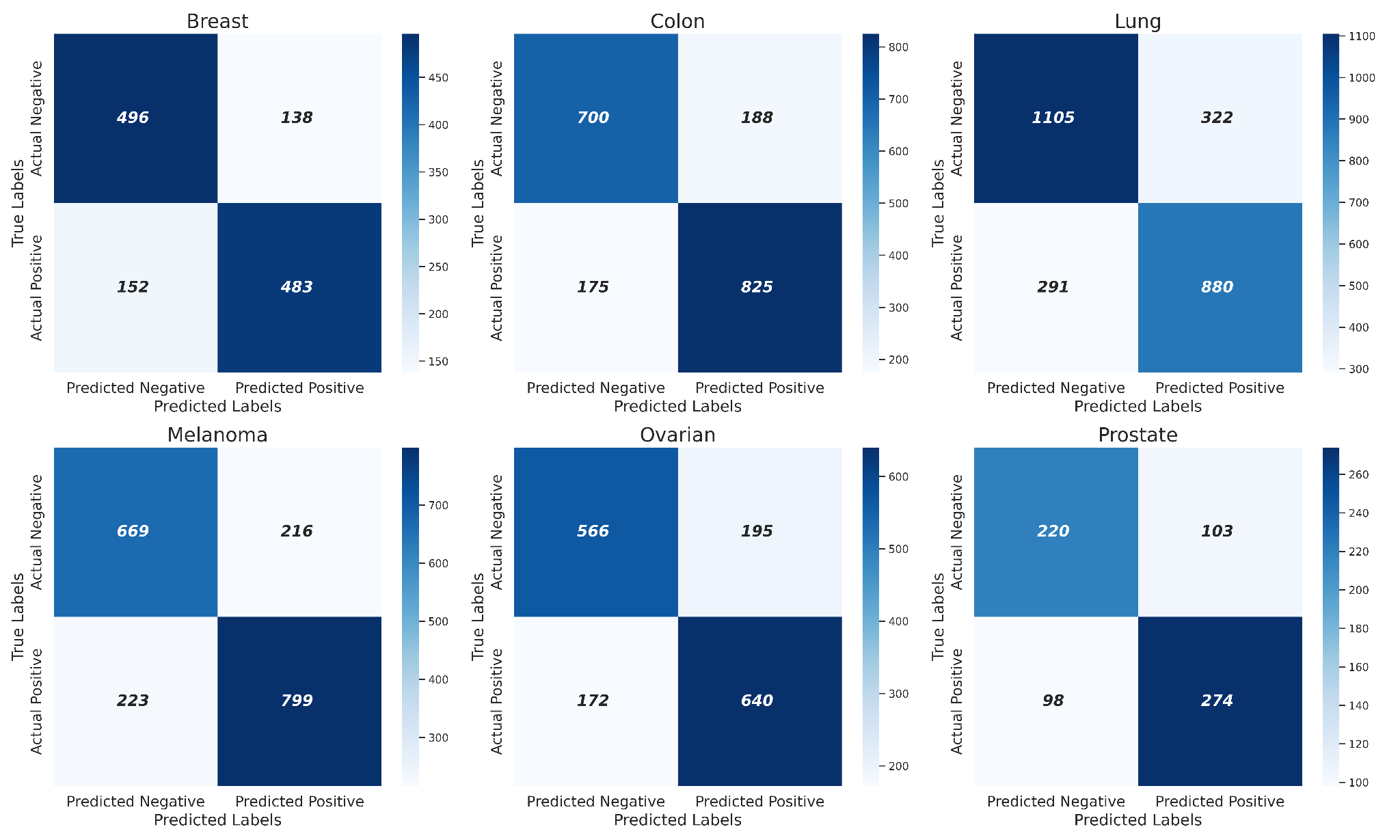
**

**FigureS6.** The confusion matrices for piscesCSM tissue-specific models on cross-validation.


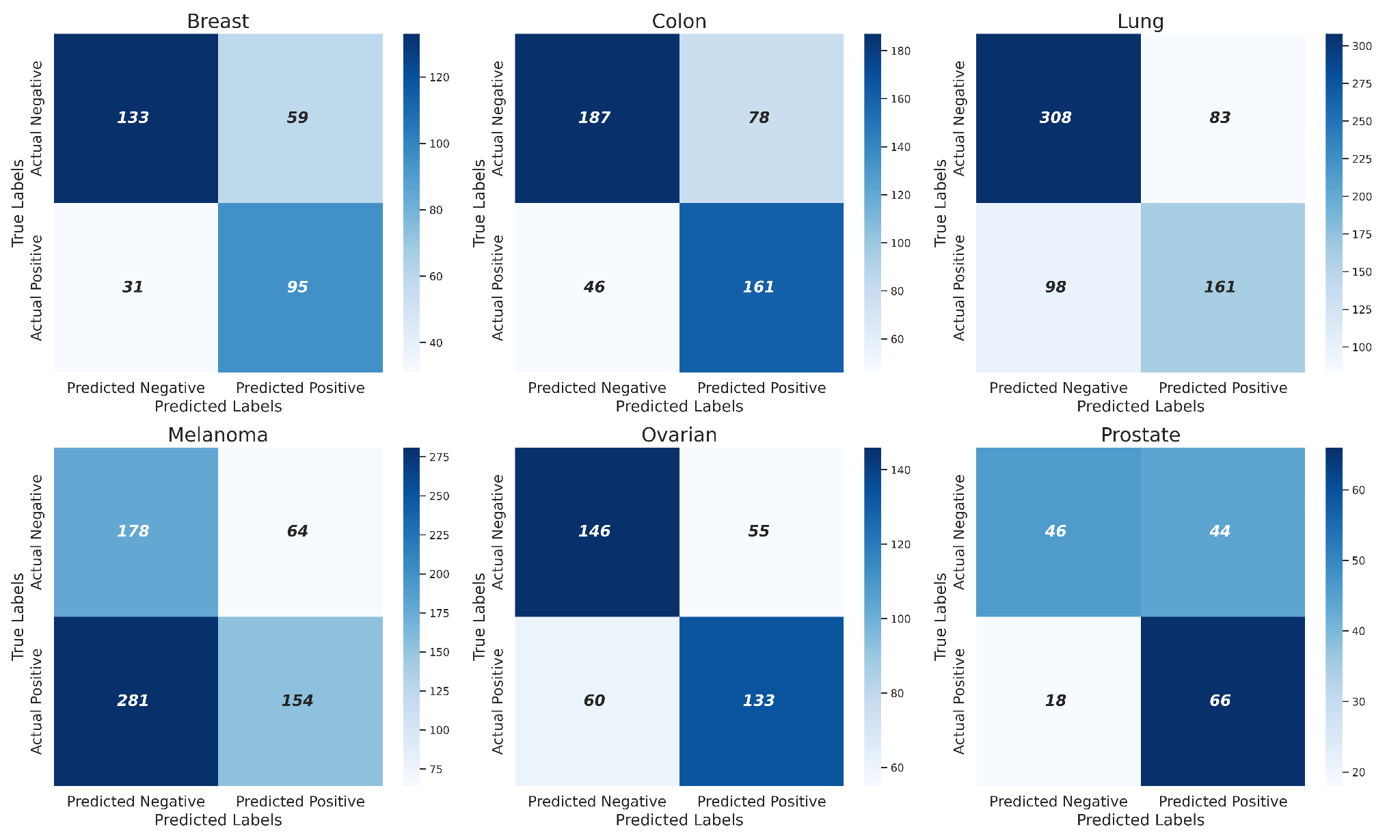


**FigureS7.** The confusion matrices for piscesCSM tissue-specific models on the blind test sets.


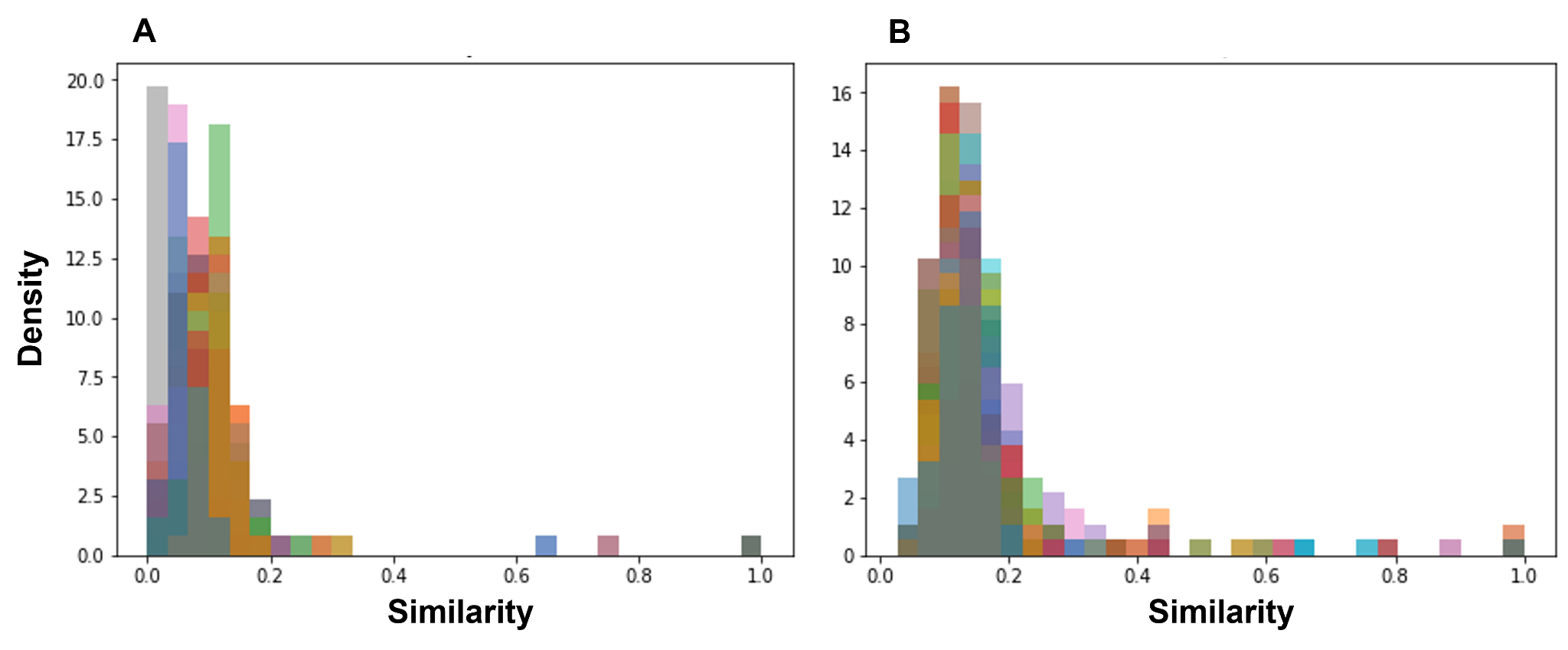


**Figure S8.** Distribution curves for Tanimoto similarity index on training (A) and AstraZeneca independent blind test sets(B).

**
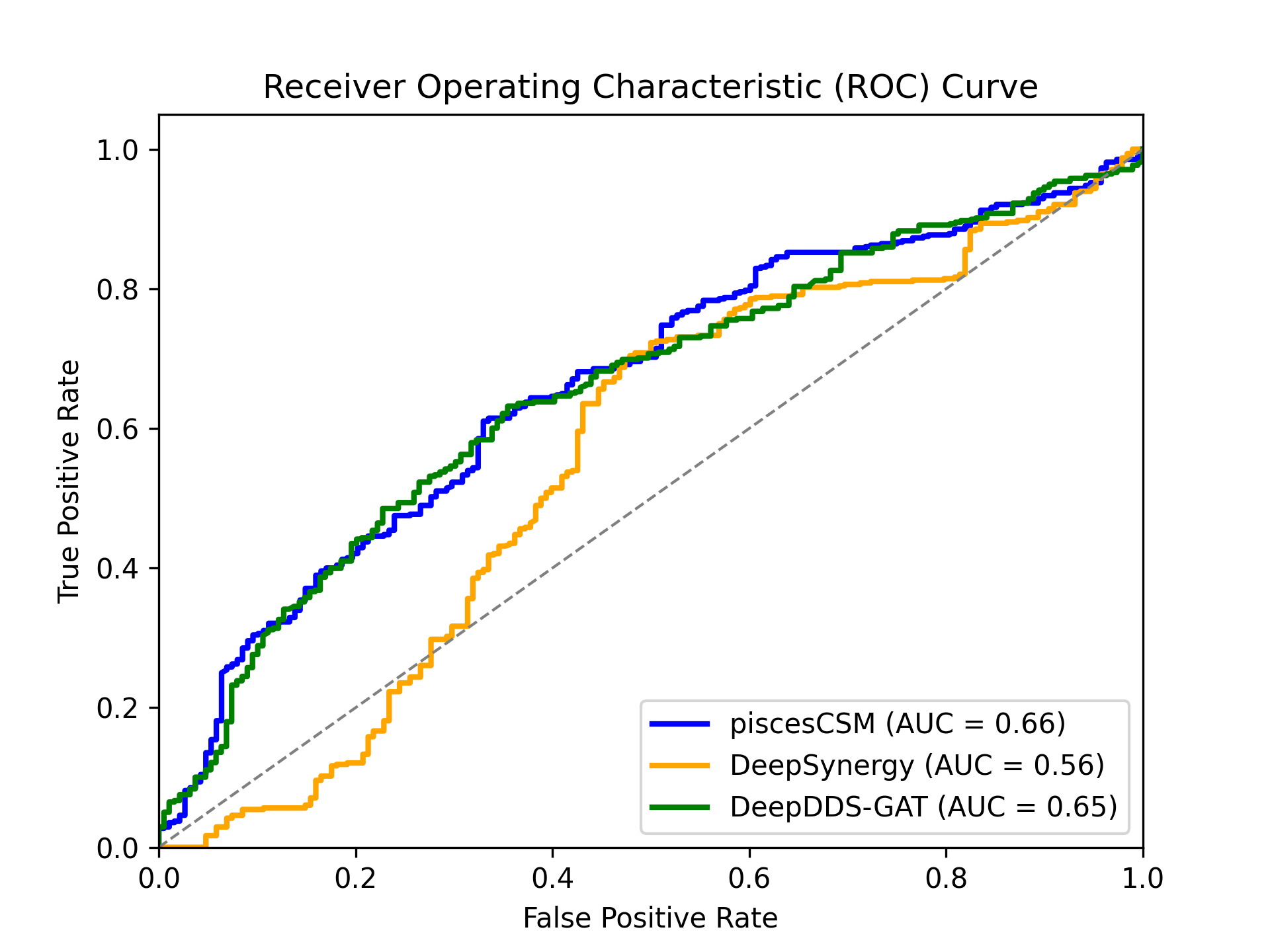
**

**Figure S9.** ROC curves of piscesCSM and the state-of-the-art methods- DeepSynergy and DeepDDS-GAT on an independent test data set published by AstraZeneca.


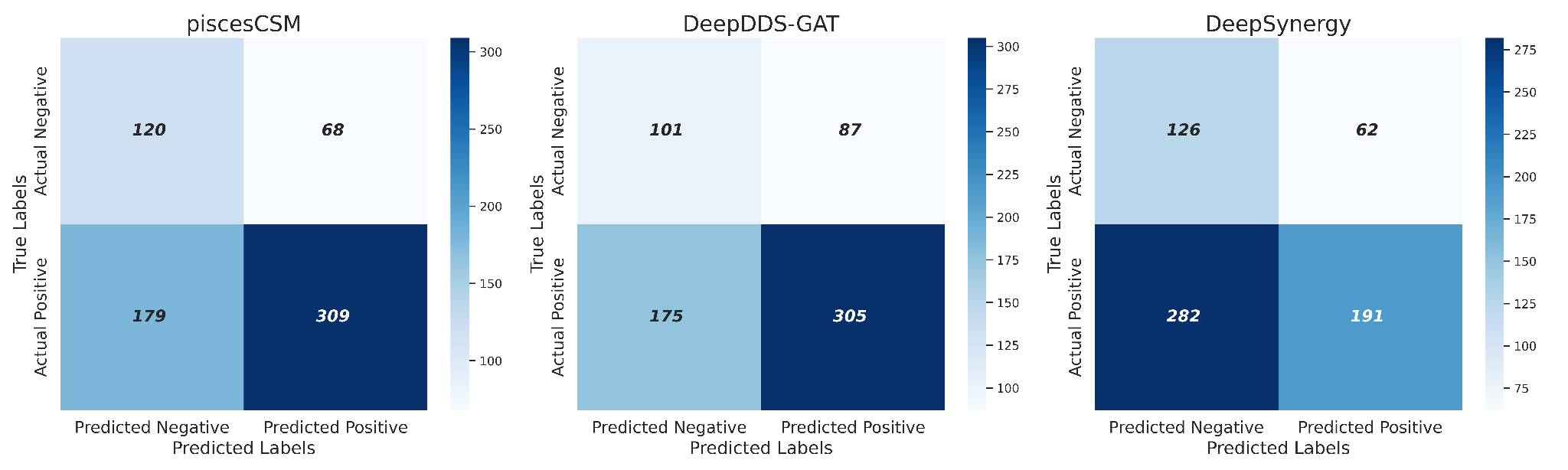


**Figure S10.** The confusion matrices of piscesCSM and the state-of-the-art methods- DeepDDS-GAT and DeepSynergy on an independent test data set published by AstraZeneca.


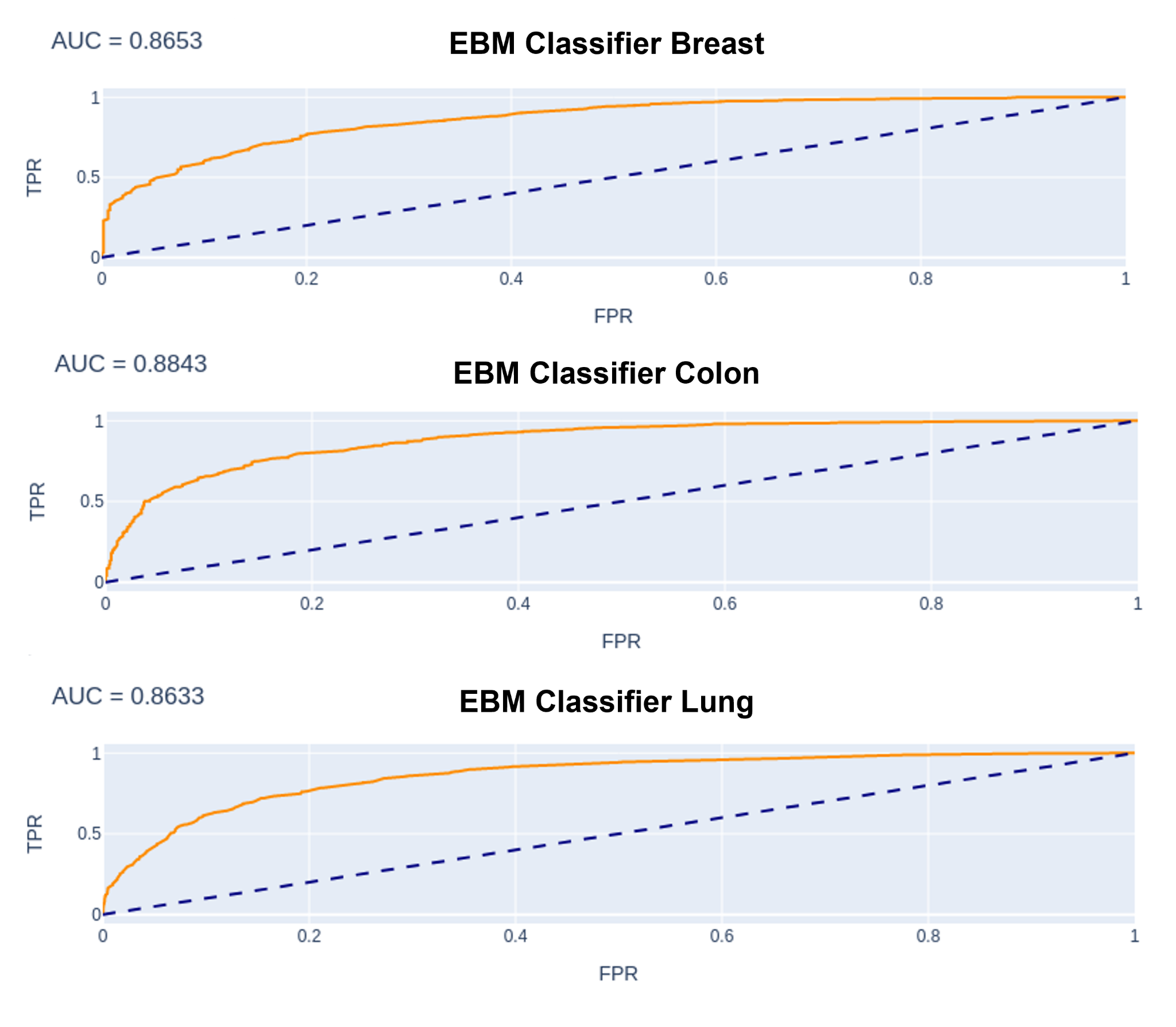


**Figure S11.** ROC Curves of the EBM-(Breast, Colon and Lung) specific classifiers on the training set.


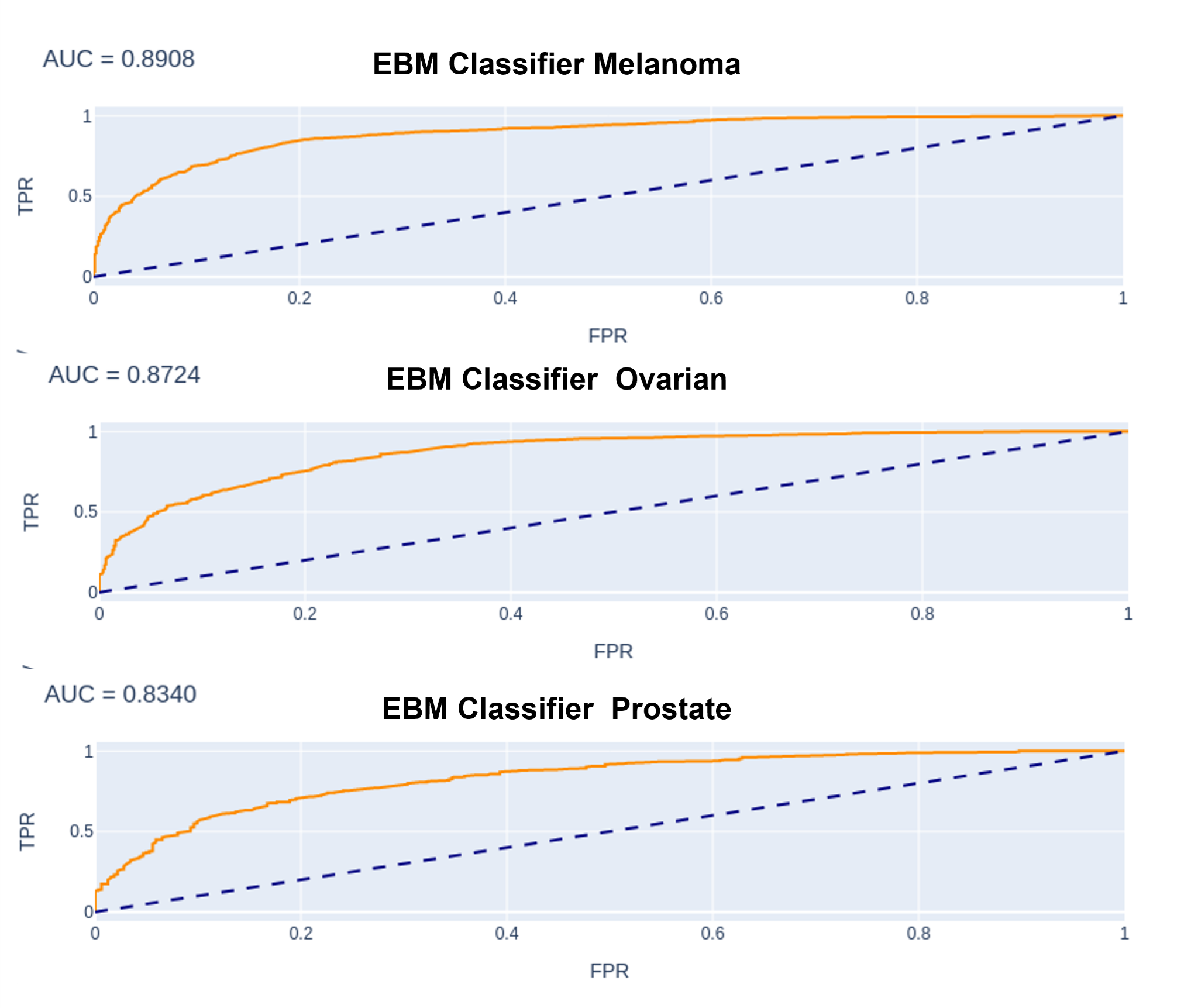


**Figure S12.**ROC Curves of the EBM-(Melanoma, Ovarian and Prostate) specific classifiers on the training set.


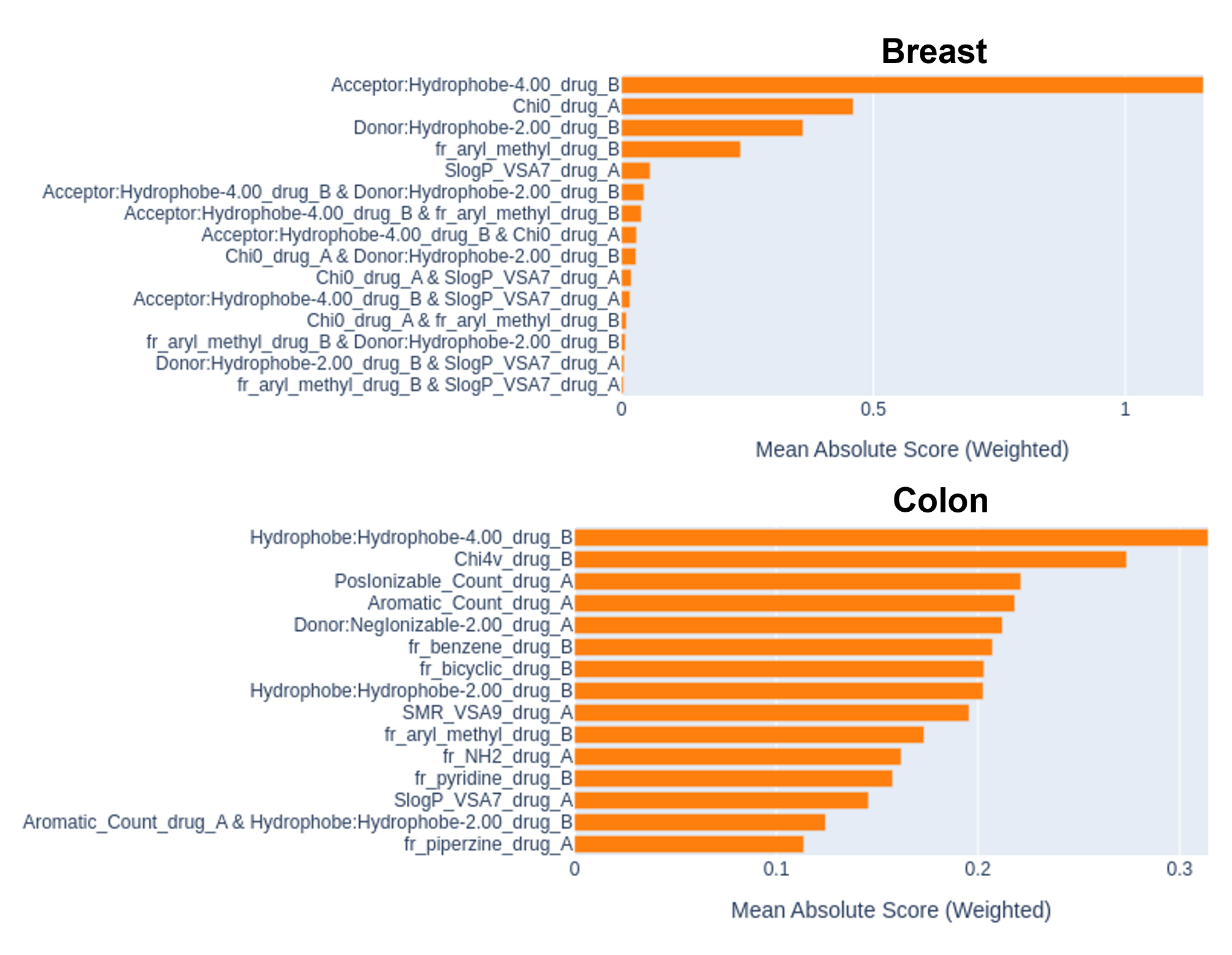


**Figure S13.** Global explanations of the Breast and Colon-specific EBM models(on the training set).


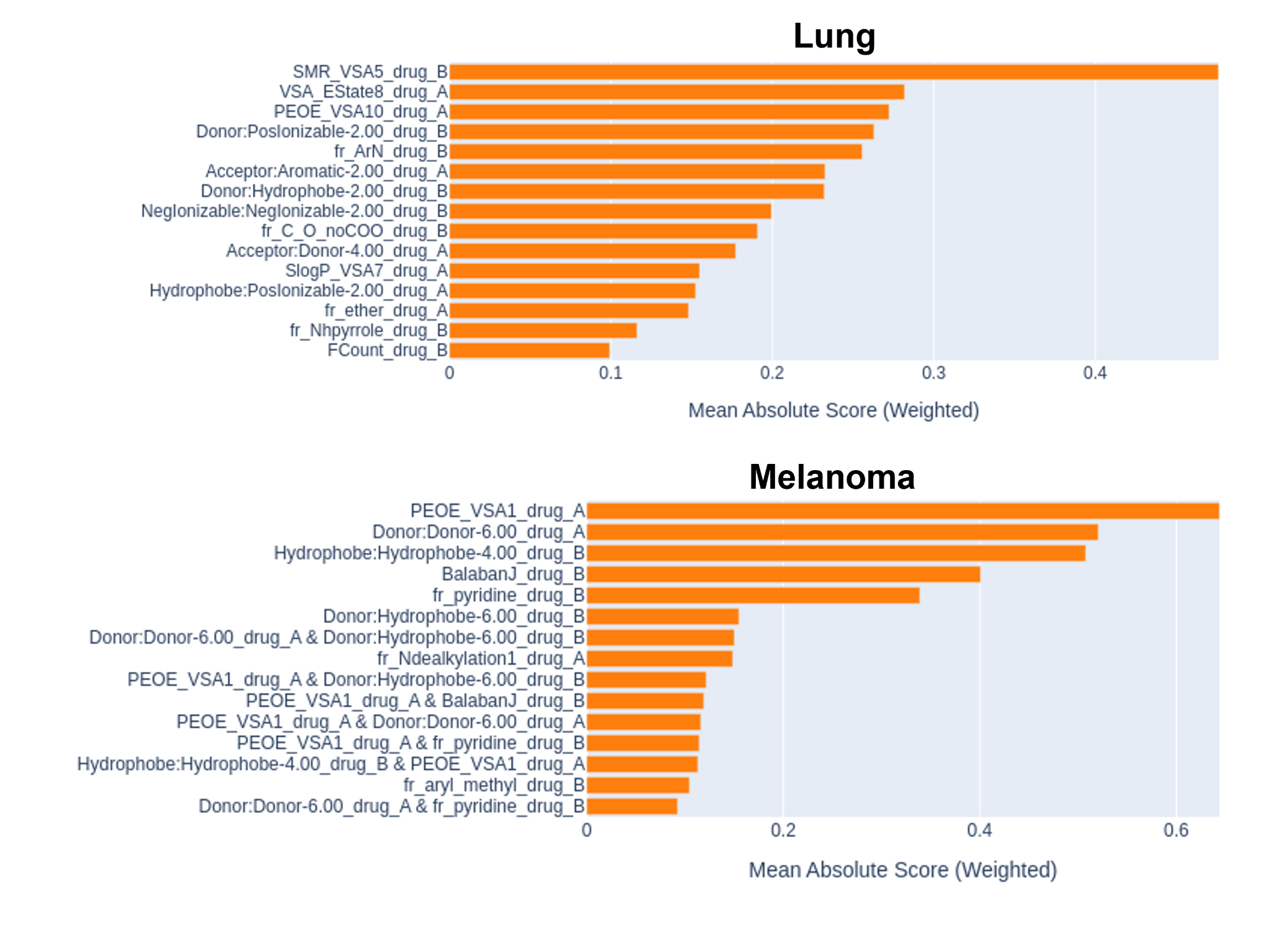


**Figure S14.** Global explanations of the Lung and Melanoma-specific EBM models (on the training set).


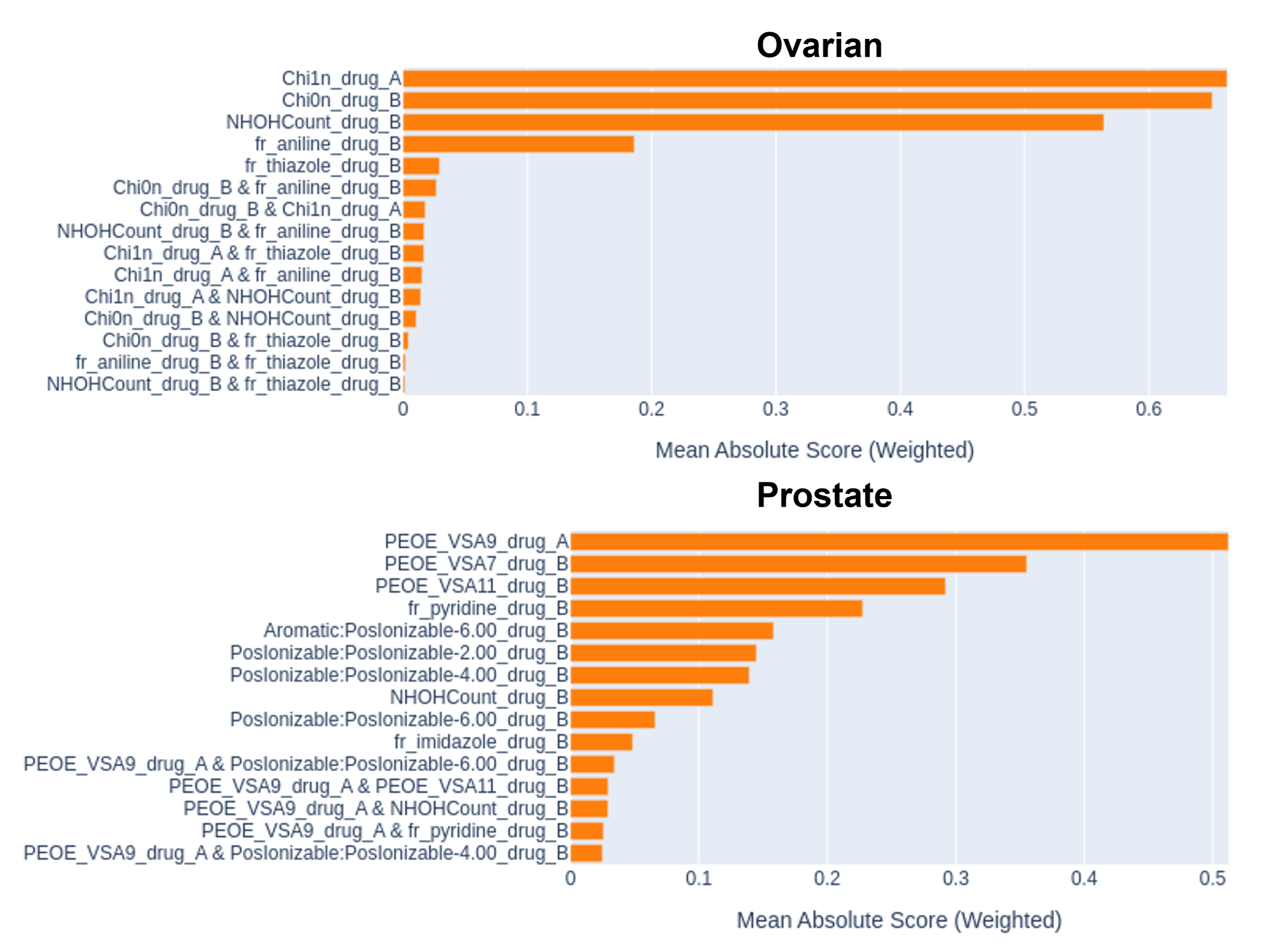


**Figure S15.** Global explanations of the Ovarian and Prostate-specific EBM models(on the training set).


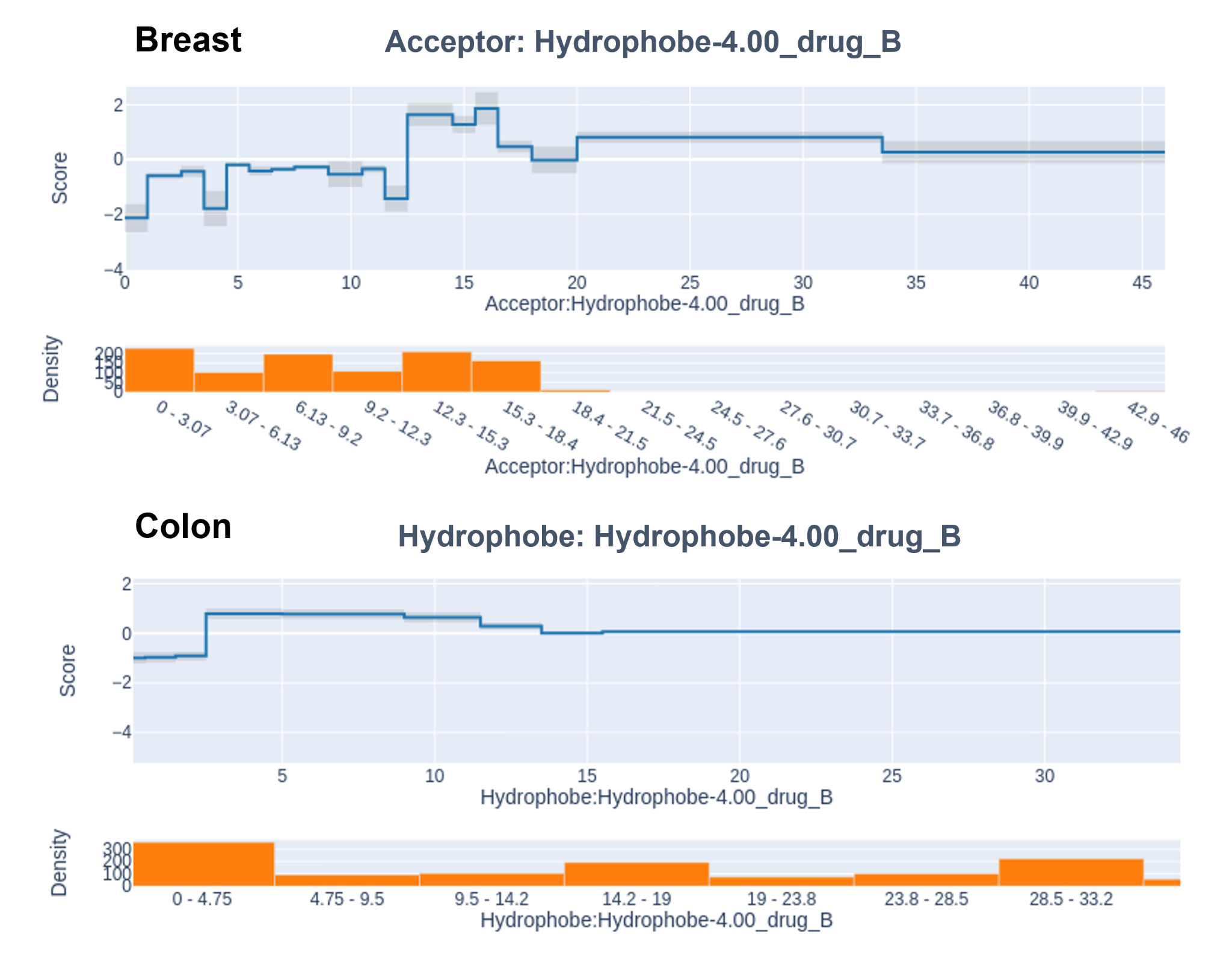


**Figure S16.** Feature interpretability plots (risk profiles) for the top most significant features in the Breast and Colon-specific models, where the upper plots show the risk score of the features, and the bottom diagrams depict the distribution of the features.


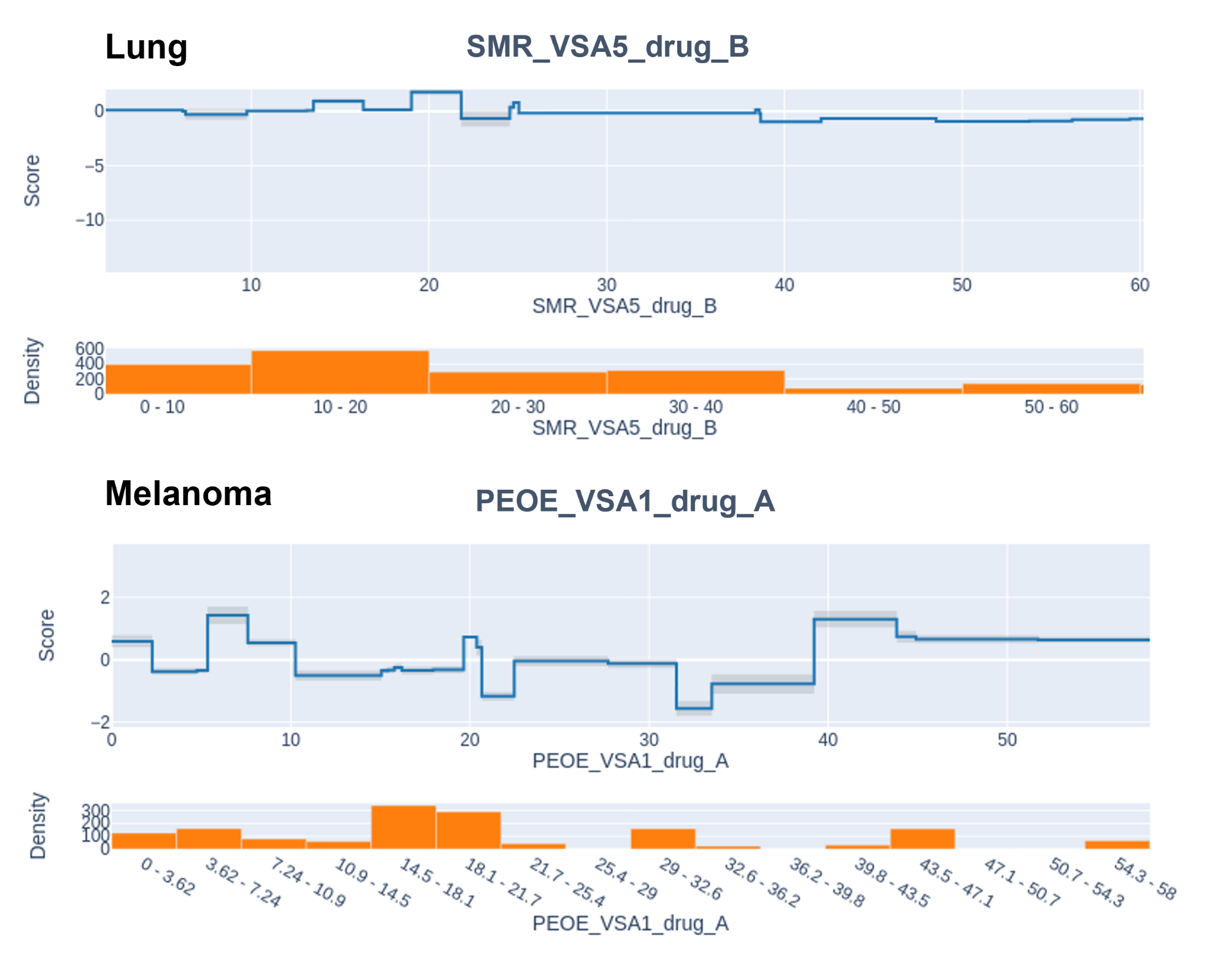


**Figure S17.** Feature interpretability plots (risk profiles) for the top most significant features in the Lung and Melanoma-specific models, where the upper plots show the risk score of the features, and the bottom diagrams depict the distribution of the features.


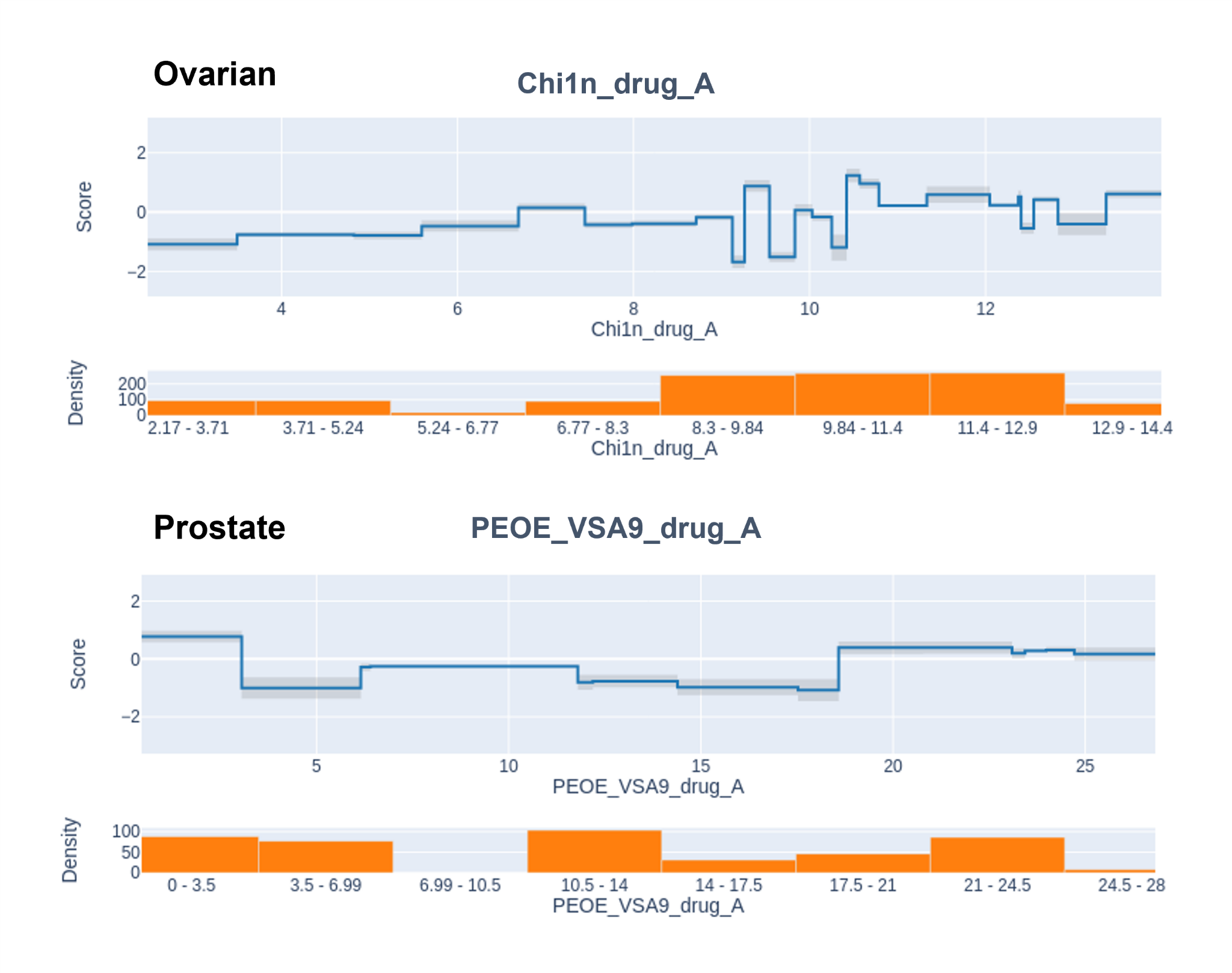


**Figure S18.**Feature interpretability plots (risk profiles) for the top most significant features in the Ovarian and Prostate-specific models, where the upper plots show the risk score of the features, and the bottom diagrams depict the distribution of the features.


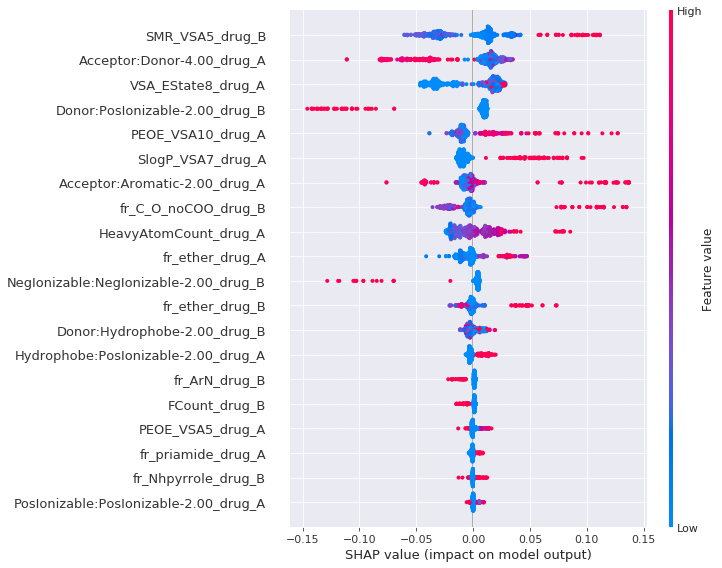


**Figure S19.** Feature importance plot of the lung tissue-specific model.
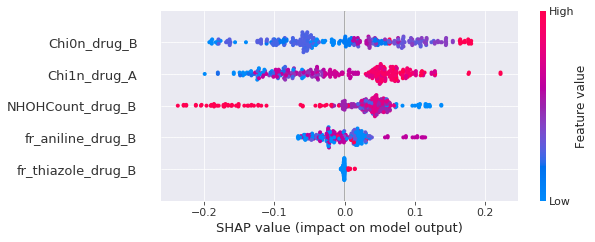


**Figure S20.** Feature importance plot of the ovarian tissue-specific model.


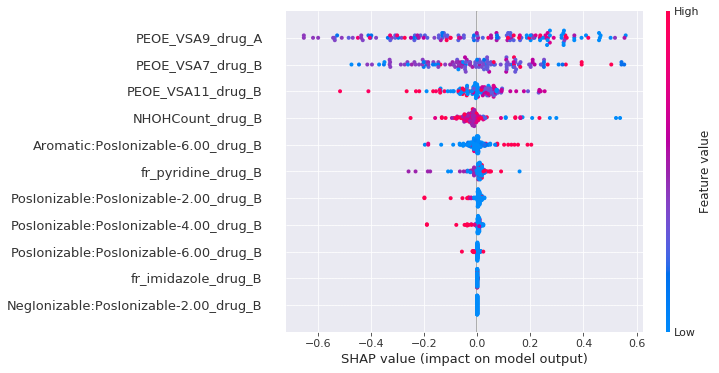


**Figure S21.** Feature importance plot of the prostate tissue-specific model.


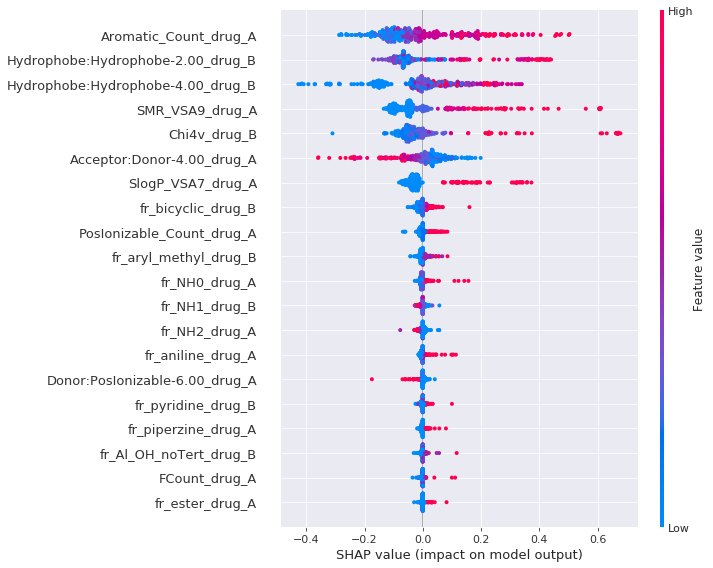


**Figure S22.** Feature importance plot of the colon tissue-specific model.
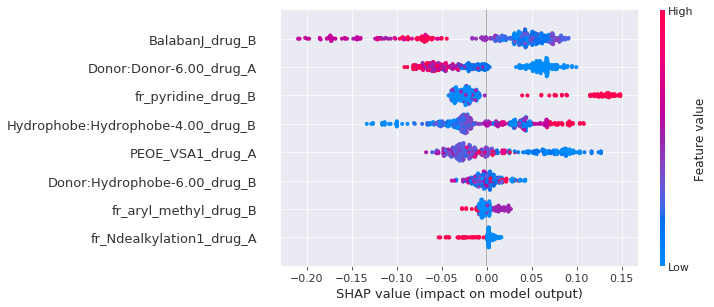


**Figure S23.** Feature importance plot of the melanoma tissue-specific model.
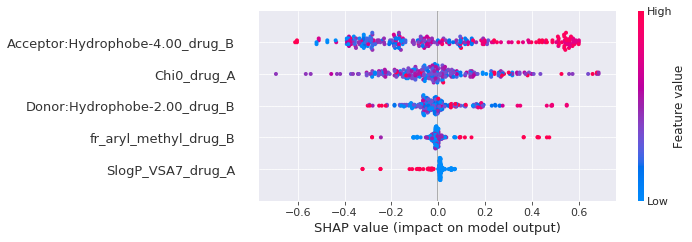


**Figure S24.** Feature importance plot of the breast tissue-specific model.


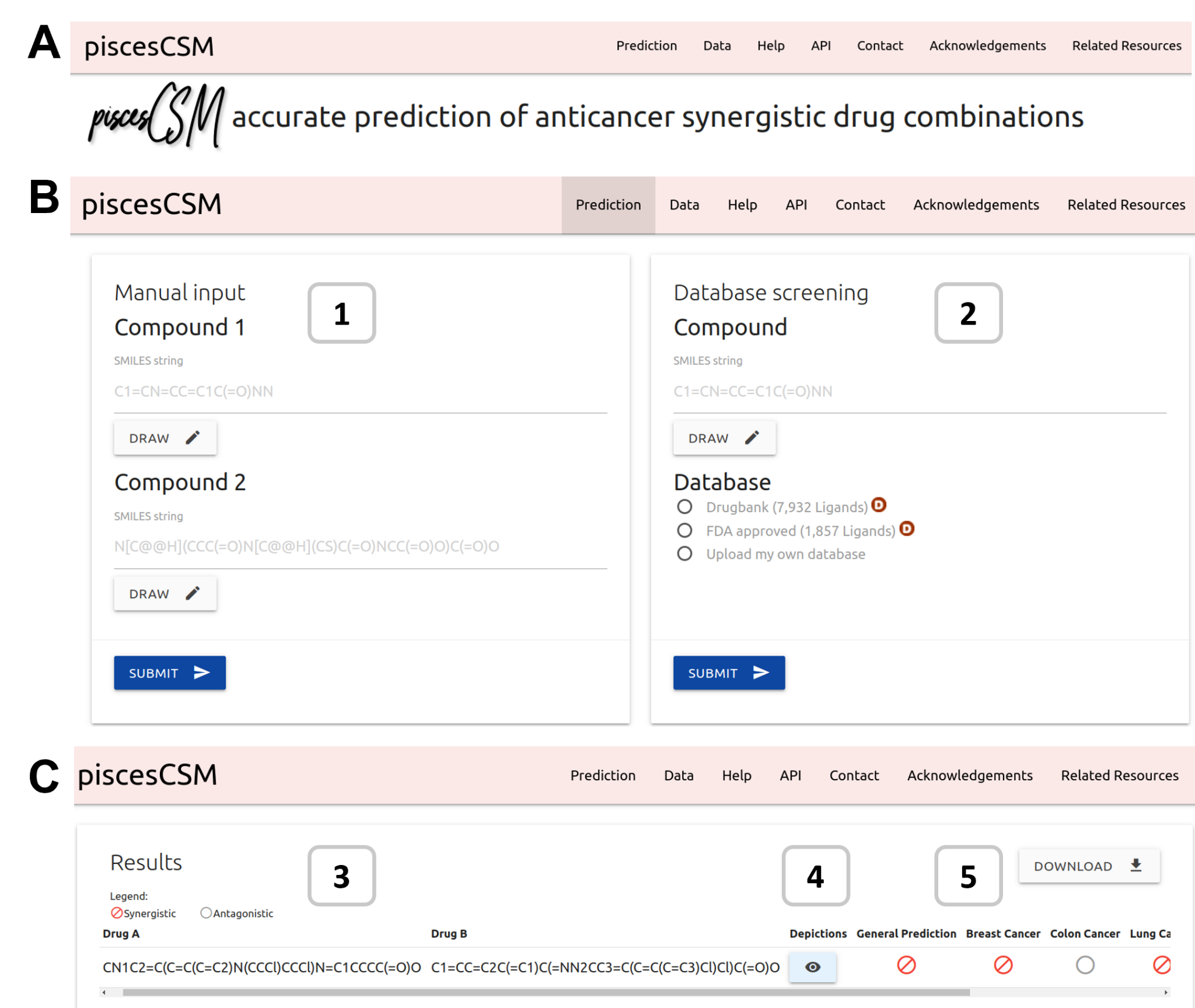


**Figure S25.** piscesCSM web server. (**A**) By clicking on "Prediction" in the top menu, users will be directed to the submission page(**B**). Users can provide their molecules (a drug pair) with two available input options: single SMILES string or molecular drawing(1). Users can also input their molecules to be screened against screening databases (2). As a result, the model predictions for synergistic effects are depicted in (**C**). The general model and six tissue model predictions are shown in tabular format(downloadable table), whether the drug pair is likely synergistic or antagonistic(3). Also, by clicking on 'Depictions' (4),piscesCSM will display molecular representations of the drug pair and pharmacokinetic properties calculated via the pkCSM tool. As a final step, users can download these results by clicking the button 'Download '(5).

**TABLES**

**Table S1.** Description of the features employed by piscesCSM to represent each small molecule.

| **General Molecular Properties** | |
| --- | --- |
| **Feature Name** | **Description** |
| HeavyAtomCount | Number of heavy atoms in the molecule |
| MolLogP | LogP value of the molecule |
| NumHeteroatoms | Number of heteroatoms in the molecule |
| NumRotatableBonds | Number of rotatable bonds in the molecule |
| RingCount | Number of rings in the molecule |
| TPSA | Topological polar surface area (TPSA) |
| LabuteASA | Labute’s approximate surface area |
| FCount | Number of fluorine atoms in the molecule |
| BalabanJ | Balaban’s J value for the molecule |
| BertzCT | Topological index quantifying molecular complexity |
| Chi0 | Molecular connectivity index (equations from [Lipkowitz and Boyd 1991]) |
| Chi0n | Modified molecular connectivity index (using nVal instead of valence) |
| Chi0v | Atomic valence connectivity index |
| Chi1 | Molecular connectivity index (equations from [Lipkowitz and Boyd 1991]) |
| Chi1n | Modified molecular connectivity index (using nVal instead of valence) |
| Chi1v | Atomic valence connectivity index |
| Chi2n | Modified molecular connectivity index (using nVal instead of valence) |
| Chi2v | Atomic valence connectivity index |
| Chi3n | Modified molecular connectivity index (using nVal instead of valence) |
| Chi3v | Atomic valence connectivity index |
| Chi4n | Modified molecular connectivity index (using nVal instead of valence) |
| Chi4v | Atomic valence connectivity index |
| HallKierAlpha | Evaluation of the Hall-Kier alpha value |
| Kappa1 | Computation of a molecular shape index |
| Kappa2 | Computation of another molecular shape index |
| Kappa3 | Computation of an additional molecular shape index |
| NHOHCount | Determination of the count of NH or OH groups in a molecule |
| NOCount | Calculation of the count of nitrogen and oxygen atoms in a molecule |
| PEOE_VSA1 | Represents MOE-type descriptors related to partial charges and surface area contributions for Charge VSA. The range is from negative infinity to less than -0.30. |
| PEOE_VSA10 | Describes MOE-type descriptors for Charge VSA with a range of 0.10 to less than 0.15. |
| PEOE_VSA11 | Refers to MOE-type descriptors for Charge VSA with a range of 0.15 to less than 0.20. |
| PEOE_VSA12 | Represents MOE-type descriptors for Charge VSA with a range of 0.20 to less than 0.25. |
| PEOE_VSA13 | Corresponds to MOE-type descriptors for Charge VSA with a range of 0.25 to less than 0.30. |
| PEOE_VSA14 | Signifies MOE-type descriptors for Charge VSA with a range of 0.30 to positive infinity. |
| PEOE_VSA2 | Indicates MOE-type descriptors for Charge VSA with a range of -0.30 to less than -0.25. |
| PEOE_VSA3 | Refers to MOE-type descriptors for Charge VSA with a range of -0.25 to less than -0.20. |
| PEOE_VSA4 | Represents MOE-type descriptors for Charge VSA with a range of -0.20 to less than -0.15. |
| PEOE_VSA5 | Describes MOE-type descriptors for Charge VSA with a range of -0.15 to less than -0.10. |
| PEOE_VSA6 | Refers to MOE-type descriptors for Charge VSA with a range of -0.10 to less than -0.05. |
| PEOE_VSA7 | Represents MOE-type descriptors for Charge VSA with a range of -0.05 to less than 0.00. |
| PEOE_VSA8 | Signifies MOE-type descriptors for Charge VSA with a range of 0.00 to less than 0.05. |
| PEOE_VSA9 | Corresponds to MOE-type descriptors for Charge VSA with a range of 0.05 to less than 0.10. |
| SMR_VSA1 | Represents MOE-type descriptors related to molar refractivity and surface area contributions for MR VSA. The range is from negative infinity to less than 1.29. |
| SMR_VSA10 | Describes MOE-type descriptors for MR VSA with a range of 1.29 to positive infinity. |
| SMR_VSA2 | MOE descriptors for molar refractivity and surface area contributions: MR VSA Descriptor 2 (1.29 <= x < 1.82) |
| SMR_VSA3 | MOE descriptors for molar refractivity and surface area contributions: MR VSA Descriptor 3 (1.82 <= x < 2.24) |
| SMR_VSA4 | MOE descriptors for molar refractivity and surface area contributions: MR VSA Descriptor 4 (2.24 <= x < 2.45) |
| SMR_VSA5 | MOE descriptors for molar refractivity and surface area contributions: MR VSA Descriptor 5 (2.45 <= x < 2.75) |
| SMR_VSA6 | MOE descriptors for molar refractivity and surface area contributions: MR VSA Descriptor 6 (2.75 <= x < 3.05) |
| SMR_VSA7 | MOE descriptors for molar refractivity and surface area contributions: MR VSA Descriptor 7 (3.05 <= x < 3.63) |
| SMR_VSA8 | MOE descriptors for molar refractivity and surface area contributions: MR VSA Descriptor 8 (3.63 <= x < 3.80) |
| SMR_VSA9 | MOE descriptors for molar refractivity and surface area contributions: MR VSA Descriptor 9 (3.80 <= x < 4.00) |
| SlogP_VSA1 | MOE descriptors for LogP and surface area contributions: LogP VSA Descriptor 1 (-inf < x < -0.40) |
| SlogP_VSA10 | MOE descriptors for LogP and surface area contributions: LogP VSA Descriptor 10 (0.40 <= x < 0.50) |
| SlogP_VSA11 | MOE descriptors for LogP and surface area contributions: LogP VSA Descriptor 11 (0.50 <= x < 0.60) |
| SlogP_VSA12 | MOE descriptors for LogP and surface area contributions: LogP VSA Descriptor 12 (0.60 <= x < inf) |
| SlogP_VSA2 | MOE descriptors for LogP and surface area contributions: LogP VSA Descriptor 2 (-0.40 <= x < -0.20) |
| SlogP_VSA3 | MOE descriptors for LogP and surface area contributions: LogP VSA Descriptor 3 (-0.20 <= x < 0.00) |
| SlogP_VSA4 | MOE descriptors for LogP and surface area contributions: LogP VSA Descriptor 4 (0.00 <= x < 0.10) |
| SlogP_VSA5 | MOE descriptors for LogP and surface area contributions: LogP VSA Descriptor 5 (0.10 <= x < 0.15) |
| SlogP_VSA6 | MOE descriptors for LogP and surface area contributions: LogP VSA Descriptor 6 (0.15 <= x < 0.20) |
| SlogP_VSA7 | MOE descriptors for LogP and surface area contributions: LogP VSA Descriptor 7 (0.20 <= x < 0.25) |
| SlogP_VSA8 | MOE descriptors for LogP and surface area contributions: LogP VSA Descriptor 8 (0.25 <= x < 0.30) |
| SlogP_VSA9 | MOE descriptors for LogP and surface area contributions: LogP VSA Descriptor 9 (0.30 <= x < 0.40) |
| VSA_EState1 | MOE descriptors for surface area contributions and EState indices: EState Descriptor 1 (-inf < x < 4.78) |
| VSA_EState10 | MOE descriptors for surface area contributions and EState indices: EState Descriptor 10 (11.00 <= x < inf) |
| VSA_EState2 | MOE descriptors for surface area contributions and EState indices: EState Descriptor 2 (4.78 <= x < 5.00) |
| VSA_EState3 | MOE descriptors for surface area contributions and EState indices: EState Descriptor 3 (5.00 <= x < 5.41) |
| VSA_EState4 | MOE descriptors for surface area contributions and EState indices: EState Descriptor 4 (5.41 <= x < 5.74) |
| VSA_EState5 | Molecular surface area contributions and EState indices: Descriptor 5 (5.74 <= x < 6.00) |
| VSA_EState6 | Molecular surface area contributions and EState indices: Descriptor 6 (6.00 <= x < 6.07) |
| VSA_EState7 | Molecular surface area contributions and EState indices: Descriptor 7 (6.07 <= x < 6.45) |
| VSA_EState8 | Molecular surface area contributions and EState indices: Descriptor 8 (6.45 <= x < 7.00) |
| VSA_EState9 | Molecular surface area contributions and EState indices: Descriptor 9 (7.00 <= x < 11.00) |
| fr_Al_COO | Count of aliphatic carboxylic acids |
| fr_Al_OH | Count of aliphatic hydroxyl groups |
| fr_Al_OH_noTert | Count of aliphatic hydroxyl groups excluding tertiary OH |
| fr_ArN | Count of nitrogen functional groups attached to aromatic rings |
| fr_Ar_COO | Count of aromatic carboxylic acids |
| fr_Ar_N | Count of aromatic nitrogen atoms |
| fr_Ar_NH | Count of aromatic amine groups |
| fr_Ar_OH | Count of aromatic hydroxyl groups |
| fr_COO | Count of carboxylic acid functional groups |
| fr_COO2 | Count of carboxylic acid functional groups (alternative definition) |
| fr_C_O | Count of carbonyl oxygen atoms |
| fr_C_O_noCOO | Count of carbonyl oxygen atoms excluding carboxylic acids |
| fr_C_S | Count of thiocarbonyl functional groups |
| fr_HOCCN | Count of specific cyclic alkyl or cyclic amines containing C(OH)CCN groups |
| fr_Imine | Count of imine functional groups |
| fr_NH0 | Count of tertiary amine functional groups |
| fr_NH1 | Count of secondary amine functional groups |
| fr_NH2 | Count of primary amine functional groups |
| fr_N_O | Count of hydroxylamine functional groups |
| fr_Ndealkylation1 | Count of groups of the form XCCNR |
| fr_Ndealkylation2 | Count of tert-alicyclic amines without heteroatoms, not quinine-like bridged N |
| fr_Nhpyrrole | Count of nitrogen atoms in H-pyrrole rings |
| fr_SH | Count of thiol functional groups |
| fr_aldehyde | Count of aldehyde functional groups |
| fr_alkyl_carbamate | Count of alkyl carbamates (susceptible to hydrolysis) |
| fr_alkyl_halide | Count of alkyl halide functional groups |
| fr_allylic_oxid | Count of allylic oxidation sites excluding steroid dienones |
| fr_amide | Count of amide functional groups |
| fr_amidine | Count of amidine functional groups |
| fr_aniline | Count of aniline functional groups |
| fr_aryl_methyl | Count of aryl methyl functional groups for potential hydroxylation |
| fr_azide | Count of azide functional groups |
| fr_azo | Count of azo functional groups |
| fr_barbitur | Count of barbiturate functional groups |
| fr_benzene | Count of benzene rings |
| fr_benzodiazepine | Count of benzodiazepine groups without additional fused rings |
| fr_bicyclic | Count of bicyclic structures |
| fr_diazo | Count of diazo functional groups |
| fr_dihydropyridine | Count of dihydropyridine functional groups |
| fr_epoxide | Count of epoxide functional groups |
| fr_ester | Count of ester functional groups |
| fr_ether | Count of ether oxygen atoms (including phenoxy groups) |
| fr_furan | Count of furan rings |
| fr_guanido | Count of guanidine functional groups |
| fr_halogen | Count of halogen atoms |
| fr_hdrzine | Count of hydrazine functional groups |
| fr_hdrzone | Count of hydrazone functional groups |
| fr_imidazole | Count of imidazole rings |
| fr_imide | Count of imide functional groups |
| fr_isocyan | Count of isocyanate functional groups |
| fr_isothiocyan | Count of isothiocyanate functional groups |
| fr_ketone | Count of ketone functional groups |
| fr_ketone_Topliss | Count of ketones excluding diaryl and α,β-unsaturated dienones, with no heteroatom on the α-carbon |
| fr_lactam | Count of β-lactam functional groups |
| fr_lactone | Count of cyclic esters (lactones) |
| fr_methoxy | Count of methoxy functional groups (-OCH₃) |
| fr_morpholine | Count of morpholine rings |
| fr_nitrile | Count of nitrile functional groups |
| fr_nitro | Count of nitro functional groups |
| fr_nitro_arom | Count of nitro groups attached to benzene rings |
| fr_nitro_arom_nonortho | Count of non-ortho nitro groups attached to benzene rings |
| fr_nitroso | Count of nitroso functional groups (excluding NO₂) |
| fr_oxazole | Count of oxazole rings |
| fr_oxime | Count of oxime functional groups |
| fr_para_hydroxylation | Count of para-hydroxylation sites |
| fr_phenol | Count of phenol functional groups |
| fr_phenol_noOrthoHbond | Count of phenolic hydroxyl groups excluding ortho intramolecular H-bond substituents |
| fr_phos_acid | Count of phosphoric acid functional groups |
| fr_phos_ester | Count of phosphoric ester functional groups |
| fr_piperdine | Count of piperidine rings |
| fr_piperzine | Count of piperazine rings |
| fr_priamide | Number of primary amides |
| fr_prisulfonamd | Number of primary sulfonamides |
| fr_pyridine | Number of pyridine rings |
| fr_quatN | Number of quarternary nitrogens |
| fr_sulfide | Number of thioether |
| fr_sulfonamd | Number of sulfonamides |
| fr_sulfone | Number of sulfone groups |
| fr_term_acetylene | Number of terminal acetylenes |
| fr_tetrazole | Number of tetrazole rings |
| fr_thiazole | Number of thiazole rings |
| fr_thiocyan | Number of thiocyanates |
| fr_thiophene | Number of thiophene rings |
| fr_unbrch_alkane | Number of unbranched alkanes of at least 4 members (excludes halogenated alkanes) |
| fr_urea | Number of urea groups |
| **Graph-based Signatures** | |
| **Feature Name** | **Description** |
| Acceptor_Count | Count of hydrogen bond acceptors |
| Aromatic_Count | Number of atoms in aromatic rings |
| Donor_Count | Count of hydrogen bond donors |
| Hydrophobe_Count | Number of hydrophobic atoms |
| NegIonizable_Count | Number of negIonazable atoms |
| PosIonizable_Count | Number of posIonazable atoms |
| Acceptor:Acceptor-6.00 | Pairs of Acceptor:Acceptor atoms within 6 bonds |
| Acceptor:Aromatic-6.00 | Pairs of Acceptor:Aromatic atoms within 6 bonds |
| Acceptor:Donor-6.00 | Pairs of Acceptor:Donor atoms within 6 bonds |
| Acceptor:Hydrophobe-6.00 | Number of pairs of Acceptor:Hydrophobe atoms within 6 bonds |
| Acceptor:NegIonizable-6.00 | Number of pairs of Acceptor:NegIonizable atoms within 6 bonds |
| Acceptor:PosIonizable-6.00 | Number of pairs of Acceptor:PosIonizable atoms within 6 bonds |
| Aromatic:Aromatic-6.00 | Number of pairs of Aromatic:Aromatic atoms within 6 bonds |
| Aromatic:NegIonizable-6.00 | Number of pairs of Aromatic:NegIonizable atoms within 6 bonds |
| Aromatic:PosIonizable-6.00 | Number of pairs of Aromatic:PosIonizable atoms within 6 bonds |
| Donor:Donor-6.00 | Number of pairs of Donor:Donor atoms within 6 bonds |
| Donor:Hydrophobe-6.00 | Number of pairs of Donor:Hydrophobe atoms within 6 bonds |
| Donor:NegIonizable-6.00 | Number of pairs of Donor:NegIonizablatoms within 6 bonds |
| Donor:PosIonizable-6.00 | Number of pairs of Donor:PosIonizable atoms within 6 bonds |
| Aromatic:Donor-6.00 | Number of pairs of Aromatic:Donor atoms within 6 bonds |
| Aromatic:Hydrophobe-6.00 | Number of pairs of Aromatic:Hydrophobe atoms within 6 bonds |
| Hydrophobe:Hydrophobe-6.00 | Number of pairs of Hydrophobe:Hydrophobe atoms within 6 bonds |
| Hydrophobe:NegIonizable-6.00 | Number of pairs of Hydrophobe:NegIonizable atoms within 6 bonds |
| Hydrophobe:PosIonizable-6.00 | Number of pairs of Hydrophobe:PosIonizable atoms within 6 bonds |
| NegIonizable:NegIonizable-6.00 | Number of pairs of NegIonizable:NegIonizable atoms within 6 bonds |
| NegIonizable:PosIonizable-6.00 | Number of pairs of NegIonizable:PosIonizable atoms within 6 bonds |
| PosIonizable:PosIonizable-6.00 | Number of pairs of PosIonizable:PosIonizable atoms within 6 bonds |
| Acceptor:Acceptor-4.00 | Number of pairs of Acceptor:Acceptor atoms within 4 bonds |
| Acceptor:Aromatic-4.00 | Number of pairs of Acceptor:Aromatic atoms within 4 bonds |
| Acceptor:Donor-4.00 | Number of pairs of Acceptor:Donor atoms within 4 bonds |
| Acceptor:Hydrophobe-4.00 | Number of pairs of Acceptor:Hydrophobe atoms within 4 bonds |
| Acceptor:NegIonizable-4.00 | Number of pairs of Acceptor:NegIonizable atoms within 4 bonds |
| Acceptor:PosIonizable-4.00 | Number of pairs of Acceptor:PosIonizable atoms within 4 bonds |
| Aromatic:Aromatic-4.00 | Number of pairs of Aromatic:Aromatic atoms within 4 bonds |
| Aromatic:Donor-4.00 | Number of pairs of Aromatic:Donor atoms within 4 bonds |
| Aromatic:Hydrophobe-4.00 | Number of pairs of Aromatic:Hydrophobe atoms within 4 bonds |
| Aromatic:NegIonizable-4.00 | Number of pairs of Aromatic:NegIonizable atoms within 4 bonds |
| Aromatic:PosIonizable-4.00 | Number of pairs of Aromatic:PosIonizable atoms within 4 bonds |
| Donor:Donor-4.00 | Number of pairs of Donor:Donor atoms within 4 bonds |
| Donor:Hydrophobe-4.00 | Number of pairs of Donor:Hydrophobe atoms within 4 bonds |
| Donor:NegIonizable-4.00 | Number of pairs of Donor:NegIonizablatoms within 4 bonds |
| Donor:PosIonizable-4.00 | Number of pairs of Donor:PosIonizable atoms within 4 bonds |
| Hydrophobe:Hydrophobe-4.00 | Number of pairs of Hydrophobe:Hydrophobe atoms within 4 bonds |
| Hydrophobe:NegIonizable-4.00 | Number of pairs of Hydrophobe:NegIonizable atoms within 4 bonds |
| Hydrophobe:PosIonizable-4.00 | Number of pairs of Hydrophobe:PosIonizable atoms within 4 bonds |
| NegIonizable:NegIonizable-4.00 | Number of pairs of NegIonizable:NegIonizable atoms within 4 bonds |
| NegIonizable:PosIonizable-4.00 | Number of pairs of NegIonizable:PosIonizable atoms within 4 bonds |
| PosIonizable:PosIonizable-4.00 | Number of pairs of PosIonizable:PosIonizable atoms within 4 bonds |
| Acceptor:Acceptor-2.00 | Number of pairs of Acceptor:Acceptor atoms within 2 bonds |
| Acceptor:Aromatic-2.00 | Number of pairs of Acceptor:Aromatic atoms within 2 bonds |
| Acceptor:Donor-2.00 | Number of pairs of Acceptor:Donor atoms within 2 bonds |
| Acceptor:Hydrophobe-2.00 | Number of pairs of Acceptor:Hydrophobe atoms within 2 bonds |
| Acceptor:NegIonizable-2.00 | Number of pairs of Acceptor:NegIonizable atoms within 2 bonds |
| Acceptor:PosIonizable-2.00 | Number of pairs of Acceptor:PosIonizable atoms within 2 bonds |
| Aromatic:Aromatic-2.00 | Number of pairs of Aromatic:Aromatic atoms within 2 bonds |
| Aromatic:Donor-2.00 | Number of pairs of Aromatic:Donor atoms within 2 bonds |
| Aromatic:Hydrophobe-2.00 | Number of pairs of Aromatic:Hydrophobe atoms within 2 bonds |
| Aromatic:NegIonizable-2.00 | Number of pairs of Aromatic:NegIonizable atoms within 2 bonds |
| Aromatic:PosIonizable-2.00 | Number of pairs of Aromatic:PosIonizable atoms within 2 bonds |
| Donor:Donor-2.00 | Number of pairs of Donor:Donor atoms within 2 bonds |
| Donor:Hydrophobe-2.00 | Number of pairs of Donor:Hydrophobe atoms within 2 bonds |
| Donor:NegIonizable-2.00 | Number of pairs of Donor:NegIonizablatoms within 2 bonds |
| Donor:PosIonizable-2.00 | Number of pairs of Donor:PosIonizable atoms within 2 bonds |
| Hydrophobe:Hydrophobe-2.00 | Number of pairs of Hydrophobe:Hydrophobe atoms within 2 bonds |
| Hydrophobe:NegIonizable-2.00 | Number of pairs of Hydrophobe:NegIonizable atoms within 2 bonds |
| Hydrophobe:PosIonizable-2.00 | Number of pairs of Hydrophobe:PosIonizable atoms within 2 bonds |
| NegIonizable:NegIonizable-2.00 | Number of pairs of NegIonizable:NegIonizable atoms within 2 bonds |
| NegIonizable:PosIonizable-2.00 | Number of pairs of NegIonizable:PosIonizable atoms within 2 bonds |
| PosIonizable:PosIonizable-2.00 | Number of pairs of PosIonizable:PosIonizable atoms within 2 bonds |

**Table S2.** Dataset Composition and Characteristics.

| **Dataset** | **Description** | **Number** |
| --- | --- | --- |
| **Combination Pairs** | Total number of drug combination pairs in the dataset | 12,415 (6,300 antagonistic and 6,115 synergistic drug pairs) |
| **Molecular Features** | Features describing the molecular properties of each drug individually | 264 |
| **Train Set Size** | Number of samples used for training the piscesCSM-model | 9,933 (5,040 antagonistic pairs /4,893 synergistic pairs) |
| **Test Set Size** | Number of samples used for testing the piscesCSM- model | 2,482 (1,260 antagonistic pairs /1,222 synergistic pairs) |
| **Total Dataset Size** | Total size of the dataset | 12, 415 |

**Table S3.** Details on hyperparameters applied in piscesCSM.

| **Model** | **Algorithm** | **Hyperparameters** | **Description** | **Value** |
| --- | --- | --- | --- | --- |
| **piscesCSM** | Extremely Randomised Trees | n_estimators | Number of decision trees in the ensemble | 400 |
|  |  | max_features | Number of input features to randomly select for each split point | auto |
|  |  | min_samples_split | Minimum number of samples required in a node to create a new split point | 2 |
|  |  | criterion | Splitting criterion | gini |
|  |  | random_state | Seed for random number generation | 1 |

**Table S4.** Predictive performance of the final model-piscesCSM before and after applying hyperparameters optimization under 5-fold cross-validation.

| **Method** | **AUC** | **MCC** | **Balanced Accuracy** | **Precision** | **Recall** |
| --- | --- | --- | --- | --- | --- |
| **Performance with tuned hyperparameters** | 0.89 | 0.61 | 0.82 | 0.82 | 0.81 |
| **Performance without tuned hyperparameters** | 0.81 | 0.57 | 0.79 | 0.79 | 0.79 |

**Table S5.**  Predictive performance of piscesCSM on 10-fold and 20-fold cross-validation.

| **Method** | **AUC** | **MCC** | **Balanced Accuracy** | **Precision** | **Recall** |
| --- | --- | --- | --- | --- | --- |
| **10-fold CV** | 0.87 | 0.61 | 0.81 | 0.81 | 0.81 |
| **20-fold CV** | 0.88 | 0.62 | 0.81 | 0.81 | 0.81 |

**Table S6.** Comparative performance of piscesCSM and other competitive methods on blind test set.

| **Method** | **AUC** | **MCC** | **Balanced Accuracy** | **Precision** | **Recall** | **F1 score** |
| --- | --- | --- | --- | --- | --- | --- |
| **piscesCSM** | 0.87 | 0.59 | 0.81 | 0.82 | 0.81 | 0.81 |
| **DeepDDS-GAT** | 0.83 | 0.58 | 0.62 | 0.80 | 0.67 | 0.73 |
| **DeepSynergy** | 0.73 | 0.42 | 0.66 | 0.64 | 0.70 | 0.67 |

**Table S7.**  A list of Training and Testing Samples by Tissue Type.

| **Tissue Type** | **Training Samples** | **Testing Samples** |
| --- | --- | --- |
| **Breast** | 1,270 (661 antagonistic, 609 synergistic) | 317 (165 antagonistic, 152 synergistic) |
| **Colon** | 1,888 (922 antagonistic, 966 synergistic) | 472 (231 antagonistic, 241 synergistic) |
| **Lung** | 2,598 (1,454 antagonistic, 1,144 synergistic) | 650 (364 antagonistic, 286 synergistic) |
| **Melanoma** | 1,908 (902 antagonistic, 1,006 synergistic) | 476 (225 antagonistic, 251 synergistic) |
| **Ovarian** | 1,574 (770 antagonistic, 804 synergistic) | 393 (192 antagonistic, 201 synergistic) |
| **Prostate** | 695 (330 antagonistic, 365 synergistic) | 174 (83 antagonistic, 91 synergistic) |

**Table S8.** Detailed information about the cell lines of an independent test set.

| **Cell line name** | **COSMIC ID** | **GDSC tissue descriptor** |
| --- | --- | --- |
| **22RV1** | 924100 | urogenital_system |
| **A549** | 905949 | lung_NSCLC |
| **C32** | 906830 | skin |
| **HCC1143** | 749710 | breast |
| **HCC1187** | 749711 | breast |
| **HCC1395** | 749712 | breast |
| **HCC1419** | 907045 | breast |
| **HCC1428** | 1290905 | breast |
| **HCC1500** | 1303900 | breast |
| **HCC1569** | 907046 | breast |
| **HCC1806** | 907047 | breast |
| **HCC1937** | 749714 | breast |
| **HCC1954** | 749709 | breast |
| **HCC38** | 749717 | breast |
| **HCC70** | 907048 | breast |
| **J82** | 753566 | urogenital_system |
| **KATOIII** | 907276 | digestive_system |
| **MCF7** | 905946 | breast |
| **RT4** | 687455 | urogenital_system |
| **SW48** | 909751 | large_intestine |
| **SW780** | 687457 | urogenital_system |
| **SW900** | 724879 | lung_NSCLC |
| **SW948** | 909757 | large_intestine |
| **TCCSUP** | 687459 | urogenital_system |
